# Improved multiparametric scrape loading-dye transfer assay for a simultaneous high-throughput analysis of gap junctional intercellular communication, cell density and viability

**DOI:** 10.1038/s41598-020-57536-3

**Published:** 2020-01-20

**Authors:** Aneta Dydowiczová, Ondřej Brózman, Pavel Babica, Iva Sovadinová

**Affiliations:** Masaryk University, Faculty of Science, RECETOX, Kamenice 5, CZ-62500 Brno, Czech Republic

**Keywords:** Cell biology, Cell signalling

## Abstract

Gap junctional intercellular communication (GJIC) is a vital cellular process required for maintenance of tissue homeostasis. *In vitro* assessment of GJIC represents valuable phenotypic endpoint that could be effectively utilized as an integral component in modern toxicity testing, drug screening or biomedical *in vitro* research. However, currently available methods for quantifying GJIC with higher-throughputs typically require specialized equipment, proprietary software and/or genetically engineered cell models. To overcome these limitations, we present here an innovative adaptation of traditional, fluorescence microscopy-based scrape loading-dye transfer (SL-DT) assay, which has been optimized to simultaneously evaluate GJIC, cell density and viability. This multiparametric method was demonstrated to be suitable for various multiwell microplate formats, which facilitates an automatized image acquisition. The assay workflow is further assisted by an open source-based software tools for batch image processing, analysis and evaluation of GJIC, cell density and viability. Our results suggest that this approach provides a simple, fast, versatile and cost effective way for *in vitro* high-throughput assessment of GJIC and other related phenotypic cellular events, which could be included into *in vitro* screening and assessment of pharmacologically and toxicologically relevant compounds.

## Introduction

Gap junctional intercellular communication (GJIC) allows an exchange of low molecular weight molecules (<1.2 kDa) between adjacent cells in both vertebrates and invertebrates^1^. GJIC is mediated through intercellular channels build from connexin proteins in vertebrates and from innexin proteins in invertebrates^1,2^. GJIC plays a central role in coordinating cell-to-cell communication and integration of signal transduction pathways controlling gene expression and cell behaviour in order to receive coordinated and collective responses from the cells across a tissue of multicellular organism^3,4^. Dysregulation of GJIC and GJIC-dependent signal integration, for example by drugs or environmental contaminants, can disrupt the normal homeostatic control of a cell behaviour and lead to numerous adverse outcomes such as cardiovascular^5^, pulmonary^6^ or reproductive system^7,8^ diseases and cancer^4,9,10^. On the other hand, timed and targeted upregulation or downregulation of GJIC, connexin channel or hemichannel activity, induced by pharmacological agents, is currently widely discussed as potential therapeutic approach for treatment of various diseases^10–12^. Accordingly, GJIC can be effectively utilized in the modern toxicological and pharmacological research, and GJIC analysis could be a valuable component of any biological study.

However, *in vitro* assessment of GJIC has been used relatively less frequently in the modern research in comparison to other and more traditional phenotypic assays, such as evaluation of cellular metabolic activity, cell proliferation, cell cycle, autophagy, apoptosis, cell migration, cytoskeletal rearangements^13^. This might be partially due to the until-recent lack of simple, rapid, accessible and affordable methods allowing *in vitro* GJIC assessment in a higher-throughput workflow. A quick and easily automated assay for quantification of GJIC would be thus very useful for many biological researchers.

The commonly used technique for *in vitro* assessment of GJIC is so-called scrape loading-dye transfer assay (SL-DT) using a membrane-impermeable and gap junction-permeable fluorescent dye, typically Lucifer Yellow (457 Da, negatively charged), which is introduced into adherent cells typically grown in a Petri dish. The frequently used protocols^14,15^ are based on the original SL-DT^16^ from 1987, only the relatively invasive scraping step was replaced by a clean-cutting with a sharp blade (e.g. scalpel). The extent of GJIC is typically quantified using image analysis to determine a control-normalized area of communicating (i.e. Lucifer Yellow-labelled) cells^14,15^. The traditional SL-DT is a low-throughput assay, typically conducted in Petri dishes, with manual or semi-automatic image acquisition and analysis. Here we present a very simple, rapid, cost-effective, robust and microplate-based assay for simultaneous one-assay evaluation of not only GJIC, but also cell density/proliferation and viability, which is possible due to introduction of additional fluorophores into the assay workflow. In contrast to the traditional assay, this innovative multiparametric version of SL-DT, coupled with (semi)-automated image acquisition and analysis for all of the evaluated parameters, allows to increase the throughput of this assay and provide much faster and less expensive approach for GJIC assessment compatible with *in vitro* high-content analysis/screening (HCA/HCS) of pharmacologically and toxicologically significant compounds.

## Results

### Innovative *vs*. traditional SL-DT assay - replacement of dextran-based fluorophores with propidium iodide

**Figure 1:**
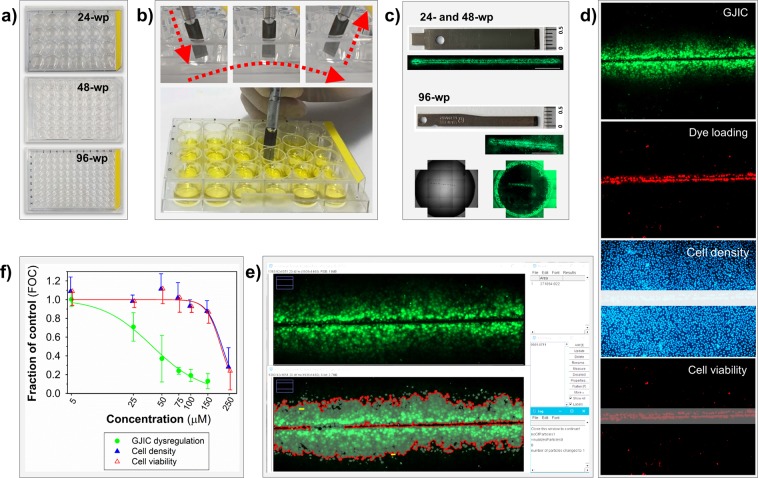
Schematic representation of (semi-) high-throughput *in vitro* multiparametric scrape-loading-dye transfer (SL-DT) assay for the simultaneous evaluation of GJIC, cell density and viability. The assay is applicable for different plastic formats (**a**–**c**) and is suitable for automatic image acquisition and analysis. The evaluation of GJIC dysregulation is based on SL-DT (**b**,**c**) using Lucifer Yellow as a marker of communicating cells (**d-GJIC**) and propidium iodide as a marker of dye-loaded cells along the cut (**d-Dye loading**). The number of total cells out of the cut is assessed by staining nuclei cell membrane permeable dye Hoechst 33342 (**d-Cell density**) and the number of dead cells out of the cut by labelling cells with propidium iodide, which is impermeable for intact, non-damaged live cells (**d-Cell viability**). The image analysis for all parameters is (semi)automatic using macros in Fiji/ImageJ (**e**). Data are expressed as fraction of control (FOC; FOC_non-treated control_ = 1.0) for GJIC, cell density and viability, as shown for a typical GJIC-inhibitory compound, 18β-glycyrrhetinic acid, which was cytotoxic at the highest concentration (**f**).

**Figure 2:**
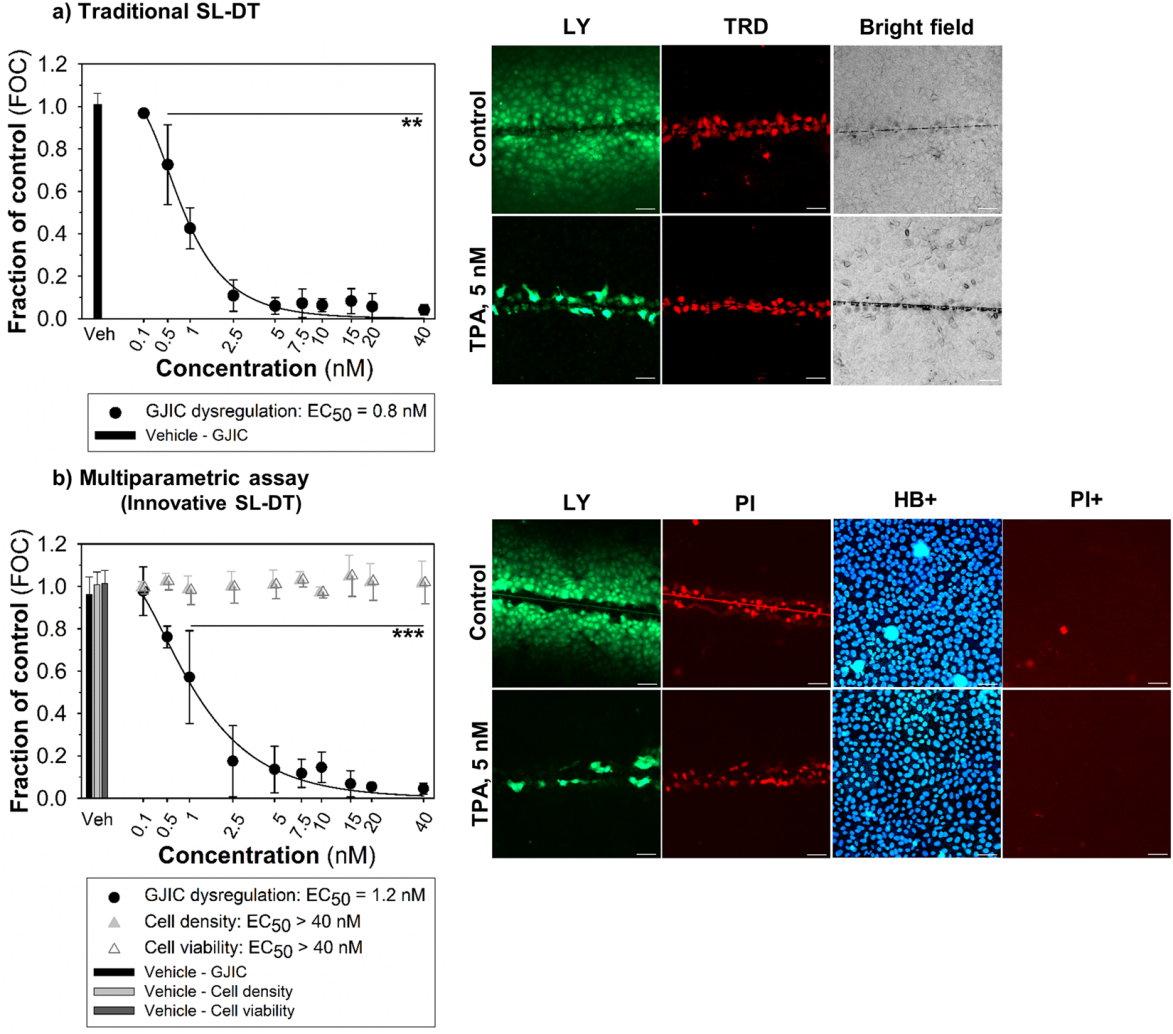
The GJIC dysregulation in rat liver progenitor WB-F344 cells induced by TPA assessed using traditional scrape-loading-dye transfer (SL-DT) assay (**a**) and innovative multiparametric SL-DT assay (**b**). Left panel: The concentration-dependent effects of TPA (0.1–40 nM) after 1-h exposure. The effects of TPA were compared with the non-treated control and expressed as the fraction of control (FOC; FOC_non-treated control_ = 1.0). Means (SD) of independent experiments are presented (n = 3). ‘**’ denote significant differences at P ≤ 0.01 (the exact P-values: 0 *vs*. 0.5 nM – 0.002; 0 *vs*. 1 to 40 nM – <0.001) and ‘***’ at P ≤ 0.001 (the exact P-values: 0 *vs*. 1 nM – 0.001; 0 *vs*. 2.5 to 40 nM – <0.001) compared to the vehicle control (one-way ANOVA with Dunnett’s test). Right panel: The representative images of communicating cells stained with Lucifer Yellow (LY), the initial loaded cells along the cut stained with Texas red-dextran (TRD) or propidium iodide (PI), the total cells stained with Hoechst 33342 (HB+) and dead cells stained with propidium iodide (PI+). TPA, 12-O-tetradecanoylphorbol-13-acetate. Bar = 50 μm.

The general schema of (semi)high-throughput *in vitro* multiparametric assay for the simultaneous evaluation of GJIC, cell density and viability with automatic image acquisition and analysis is outlined in Fig. 1. As in the traditional SL-DT method, the GJIC evaluation in the innovative multiparametric assay is based on the ability of small dyes, such as Lucifer Yellow, to diffuse from the dye-loaded cells into adjacent ones through functional gap junction channels (Fig. 1d-GJIC). To determine, which cells were initially loaded after the cut with a steel blade, and verify, that a dye transfer occurs through intercellular gap junctions, the traditional SL-DT uses another membrane impermeable fluorescent dye, such as Texas red-dextran (MW 10,000), concurrently with the gap junction diffusional dye. Texas red-dextran is too large molecule to traverse the gap junction channels, hence it is accumulated in the initially loaded cells along the scalpel cut. The area of the initially loaded, dextran-labelled cells is subtracted from the area of Lucifer Yellow-stained cells for each evaluated cut region to obtain the net area of Lucifer Yellow dye transfer via GJIC. Our innovative SL-DT assay uses propidium iodide as a marker of the dye-loaded cells (Fig. 1d – Dye loading) instead of dextran-based fluorophores. Propidium iodide is considerably less expensive than Texas red-dextran, it can be used at a lower concentration (10 μg/mL *vs* 5 mg/mL), and it can be re-used several times (Supplementary Table S1). The substitution of Texas red-dextran for propidium iodide was evaluated first using rat liver progenitor cells WB-F344 grown in 35-mm dishes (Fig. 2). The stained areas of the dye-loaded cells were comparable between Texas red-dextran (e.g. 80,021 ± 23,115 μm^2^ for a non-treated control per single field of view, FOV = 1396 × 1053 μm) and propidium iodide (e.g. 96,748 ± 6,039 μm^2^ for a non-treated control per FOV). In addition, the substitution did not change the evaluation of cellular response to a well-known GJIC inhibitor, TPA (12-O-tetradecanoylphorbol-13-acetate). In both SL-DT set-ups, TPA induced a clear concentration-dependent dysregulation of GJIC after 1-h exposure with comparable and statistically not different EC_50_ values (EC_50_ value: for the traditional SL-DT assay = 0.8 nM, for the innovative SL-DT = 1.2 nM; two-tailed t-test, P = 0.337; Supplementary Fig. S1).

### Innovative SL-DT assay is multiparametric and suitable for different formats of cell culture plasticware

The SL-DT assay has been traditionally conducted in Petri dishes, which limited its speed and throughput. Therefore, surgical scalpels typically used for dye-loading in traditional SL-DT assay were replaced in this study with a commercially available type of micro knives, which allowed to conduct the innovative SL-DT assay in different formats of cell culture plasticware – individual Petri dishes as well as different types of microplates (24-, 48-, 96-well). The average ± S.D. length of the cut was app. 5,760 ± 170 μm for steel blade #17 and app. 2,770 ± 40 μm for steel blade #66, respectively (Supplementary Fig. S2). The dye-transfer in monolayer of non-treated cells loaded with the larger blade was quite uniform along middle, app. 5,000 μm long, section of the individual cut. The average ± S.D. area of Lucifer Yellow staining for different FOVs obtained from different parts of the single cut were 436,200 ± 17,220 μm^2^ for a 35-mm Petri dish, 458,080 ± 31,440 μm^2^ for a 24-well plate, 529,120 ± 16,540 μm^2^ for a 48-well plate, with coefficients of variance less than 10%. The comparable value (564,660 μm^2^) was obtained also for a 96-well plate, where the smaller blade allowed to acquire only a single 1396 × 1053 μm FOV from the uninterrupted middle part of the cut. FOVs capturing the ends of the cuts were not included into standardized evaluation of dye transfer area, since they cover only shorter cut sections. Also, the peripheral parts of the cut appeared to be more prone to various artefacts and irregularities (scratching, cell peeling) (Supplementary Fig. S2), probably resulting from variably larger pressure applied on the tip of the blade.

To demonstrate versatility of the multiparametric SL-DT assay, we tested also other fixable polar tracers excitable at ~488 nm, such as calcein, FITC and CF™ 488A hydrazide, for assessment of gap junction functionality in our protocol (Supplementary Fig. S3a). These dyes differ in their molecular weight and size of their negative charge (see Material and Methods). In experiments with WB-F344 cells, the greatest dye-transfer areas were obtained with Lucifer Yellow (the area of Lucifer Yellow- *vs*. propidium iodide-stained cells per FOV: 492,260 ± 27,280 μm^2^ *vs*. 72,480 ± 11,690 μm^2^), followed by CF™ 488A (the area of CF™ 488A- *vs*. propidium iodide-stained cells per FOV: 234,520 ± 5,440 μm^2^ *vs*. 79,470 ± 18,120 μm^2^), and calcein (the area of calcein- *vs*. propidium iodide-stained cells per FOV: 199,230 ± 27,280 μm^2^ *vs*. 75,190 ± 23,760 μm^2^). While FITC staining exhibited the brightest fluorescent signal (the exposure time was 15-times lower than for the other dyes), the dye had only limited diffusion via gap junctions (the area of FITC- *vs*. propidium iodide-stained cells per FOV: 135,440 ± 40,310 μm^2^ *vs*. 69,280 ± 17,650 μm^2^), and also high levels of binding to the cells outside the cut area, which increased the image background. Although all four dyes were capable to detect also TPA-induced inhibition of GJIC (Supplementary Fig. S3b), CF^TM^ 488A or calcein exhibited the largest dye-transfer next to Lucifer Yellow, and produced also high contrast images, and represent examples of alternative gap junction tracers suitable for multiparametric SL-DT assay.

**Figure 3:**
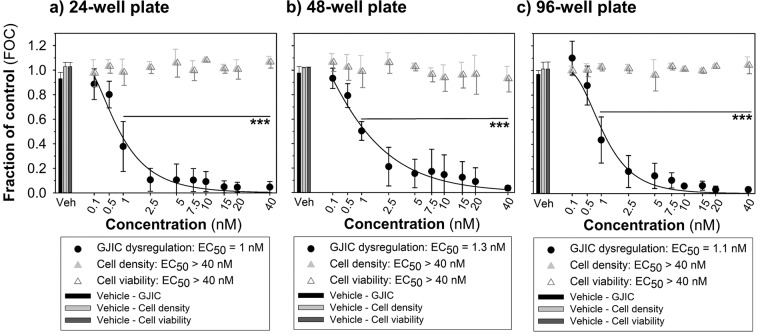
The GJIC dysregulation and the effect on cell density and viability in liver progenitor cells WB-F344 caused by TPA assessed using the multiparametric scrape-loading-dye transfer (SL-DT) assay in a variety of cell culture plastic formats – (**a**) 24-well plate, (**b**) 48-well plate, (**c**) 96 well-plate (1-h exposure). The effects of TPA were compared with the non-treated control and expressed as the fraction of control (FOC; FOC_non-treated control_ = 1.0). Means (SD) of independent experiments are presented (n = 3). ‘***’ denote significant differences at P ≤ 0.001 (the exact P-values: 0 *vs*. 1 to 40 nM – <0.001) compared to the vehicle control (one-way ANOVA with Dunnett’s test). TPA, 12-O-tetradecanoylphorbol-13-acetate.

The response of WB-F344 cells to TPA exposure (1 h) was comparable in all used types of plasticware formats (EC_50_ values: for a 35-mm Petri dish = 1.2 nM; for a 24-well plate = 1 nM; for a 48-well plate = 1.3 nM; for a 96-well plate = 1.1 nM; Figs. 2 and 3) with no statistically significant differences (one-way ANOVA, P = 0.750; Supplementary Fig. S1). In addition to assessment of GJIC dysregulation, our innovative multiparametric assay allows to evaluate also additional cellular endpoints, such as cell density and viability (Fig. 1d-Cell density and Cell viability). Both parameters were evaluated in the area outside of the dye-loading cut. Cell density was quantified by staining of cell nuclei with Hoechst 33342; number of viable cells was acquired by subtracting the number of dead cells stained with propidium iodide. TPA inhibited GJIC in WB-F344 cells practically completely at concentrations >2.5 nM without any observable effects on cell density or viability up to 40 nM (Figs. 2 and 3).

### Multiparametric SL-DT assay is compatible with (semi-)automated image acquisition and analysis

**Figure 4:**
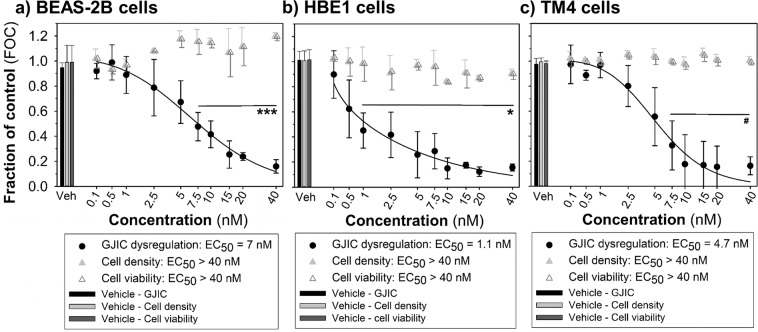
The GJIC dysregulation and the effect on cell density and viability caused by TPA assessed using the multiparametric scrape-loading-dye transfer (SL-DT) assay (a 24-well plate format) in a variety of cell types – human bronchial epithelial BEAS-2B cells (**a**) and HBE1 cells (**b**), and murine Sertoli TM4 cells (**c**). Exposure times: 1 h (TM4 cells), 3 h (BEAS-2B) or 24 h (HBE1). The effects of TPA were compared with the non-treated control and expressed as the fraction of control (FOC; FOC_non-treated control_ = 1.0). Means (SD) of independent experiments are presented (n = 3). ‘*’ denotes significant differences at P ≤ 0.05 (the exact P-values: 0 *vs*. 0.5 nM – 0.017, 0 *vs*. 1 to 40 nM – <0.001) and ‘***’ at P ≤ 0.001 (the exact P-values: 0 *vs*. 7.5 to 40 nM – <0.001) compared to the vehicle control (one-way ANOVA with Dunnett’s test). ‘#’ denotes significant differences at P ≤ 0.05 compared to the vehicle control (Kruskal-Wallis ANOVA with Dunnett’s test). TPA, 12-O-tetradecanoylphorbol-13-acetate.

**Figure 5:**
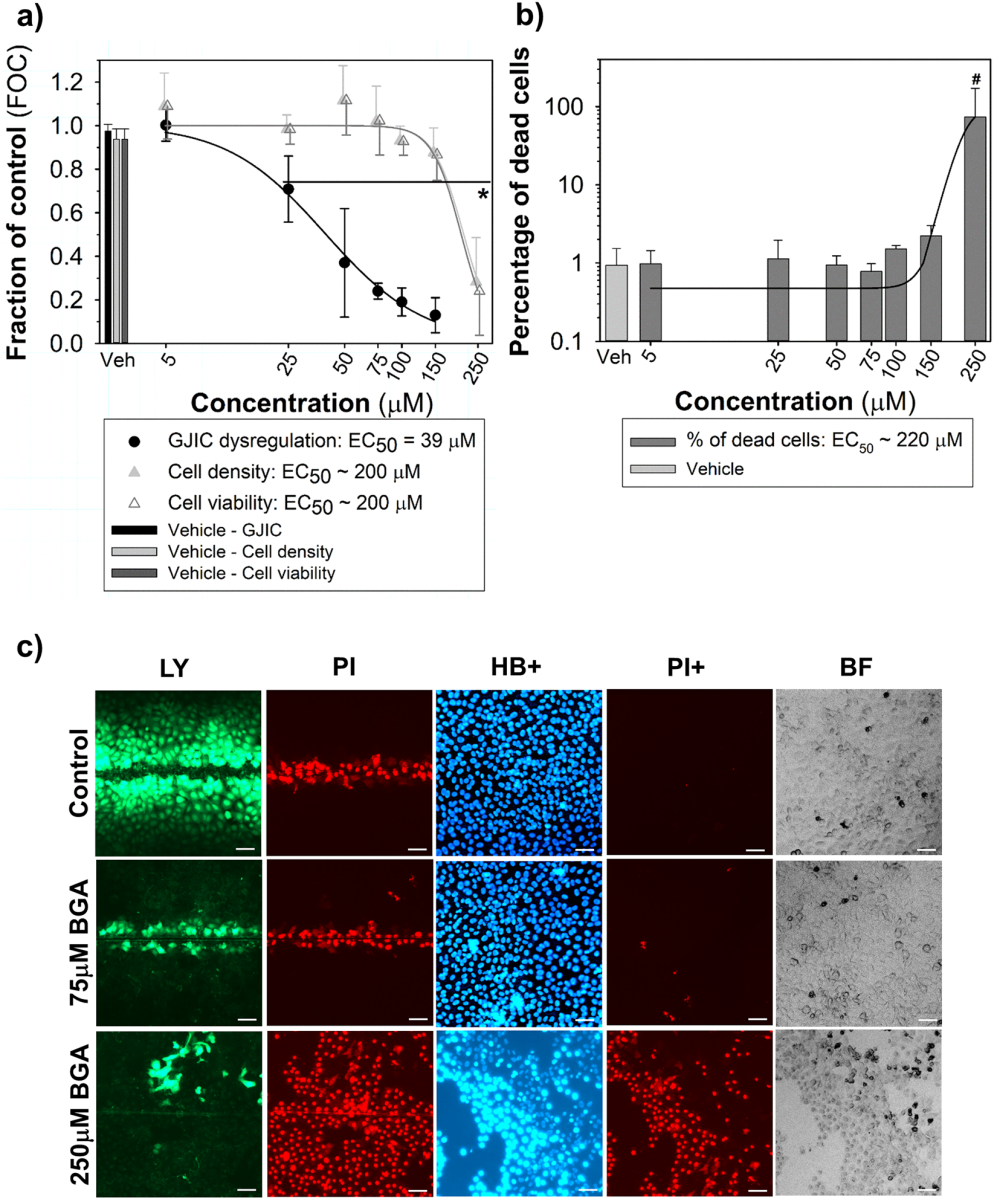
18-β-glycyrrhetinic acid as a model GJIC-inhibitor assessed using the multiparametric scrape-loading-dye transfer (SL-DT) assay in rat liver progenitor WB-F344 cells (a 24-well plate format, 2-h exposure). (**a**) The effects were compared with the non-treated control and expressed as the fraction of control (FOC; FOC_non-treated control_ = 1.0). Means (SD) of independent experiments are presented (n = 3). ‘*’ denotes significant differences at P ≤ 0.05 (the exact P-values for GJIC: 0 *vs*. 25 μM – 0.045, 0 *vs*. 50 to 200 μM – <0.001; for density and viability: 0 *vs*. 200 μM – <0.001) compared to the vehicle control (one-way ANOVA with Dunnett’s test). (**b**) The % of dead cells were calculated as % of propidium iodide (PI)-stained cells compared to Hoechst 33342 (HB)-stained cells. Means (SD) of independent experiments are presented (n = 3). The “#” denotes significant differences at P ≤ 0.05 compared to the vehicle control (Kruskal-Wallis ANOVA with Dunnett’s test). (**c**) The representative images of communicating cells stained with Lucifer Yellow (LY), the initial loaded cells along the cut stained with propidium iodide (PI), the total cells stained with Hoechst 33342 (HB+) and dead cells stained with PI (PI+). Bar = 50 μm.

**Figure 6:**
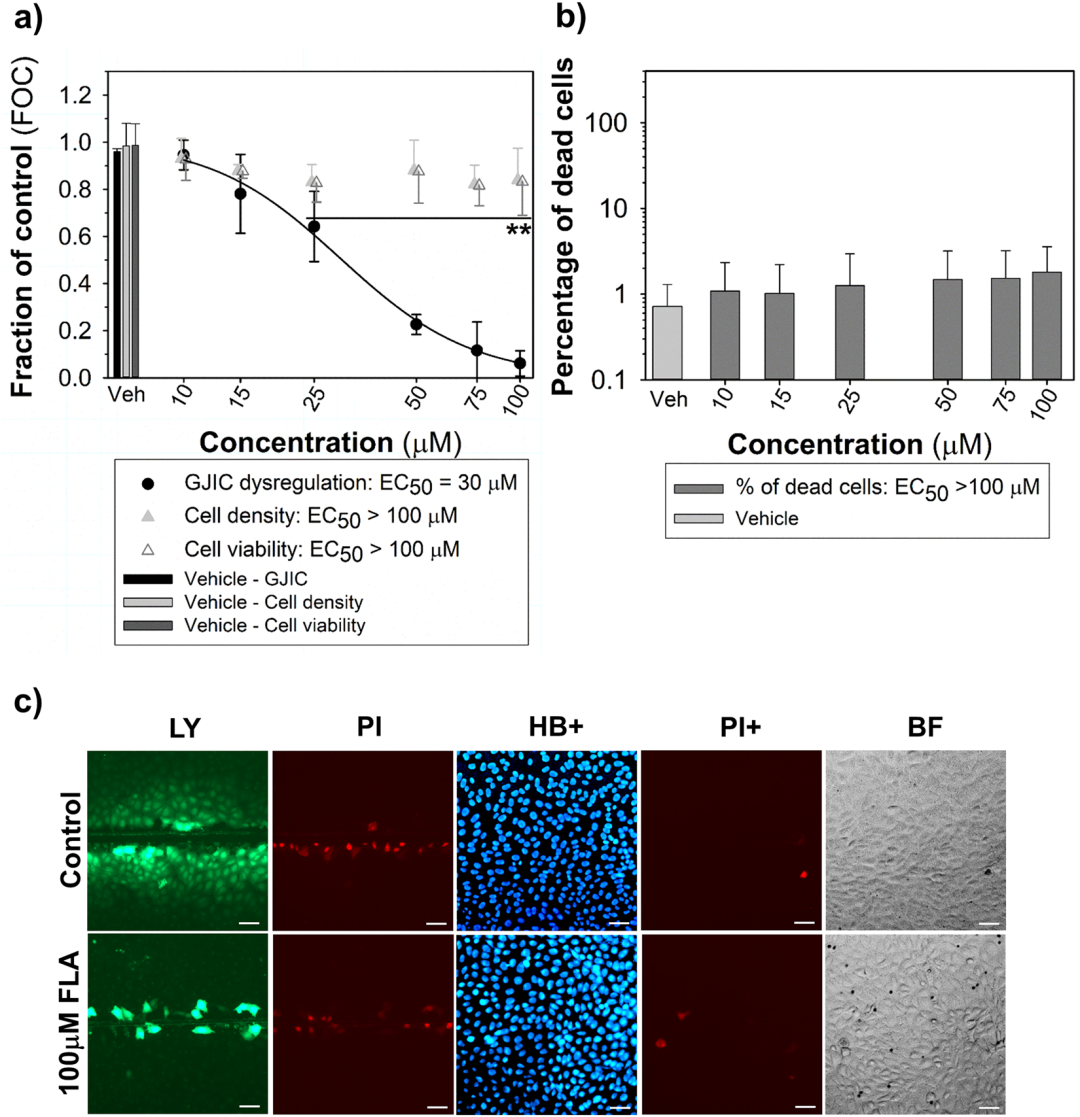
Fluoranthene as a non-cytotoxic GJIC inhibitor assessed using the multiparametric scrape-loading-dye transfer (SL-DT) assay in rat liver progenitor WB-F344 cells (a 24-well plate format, 1-h exposure). (**a**) The effects were compared with the non-treated control and expressed as the fraction of control (FOC; FOC_non-treated control_ = 1.0). Means (SD) of independent experiments are presented (n = 3). ‘**’ denote significant differences at P ≤ 0.01 (the exact P-values: 0 *vs*. 25 μM – 0.010, 0 *vs*. 50 to 100 μM – <0.001) compared to the vehicle control (one-way ANOVA with Dunnett’s test). (**b**) The % of dead cells were calculated as % of propidium iodide (PI)-stained cells compared to Hoechst 33342 (HB)-stained cells. Means (SD) of independent experiments are presented (n = 3). No significant differences compared to the vehicle control (P > 0.05; one-way ANOVA). (**C**) The representative images of communicating cells stained with Lucifer Yellow (LY), the initial loaded cells along the cut stained with propidium iodide (PI), the total cells stained with Hoechst 33342 (HB+) and dead cells stained with PI (PI+). Bar = 50 μm.

**Figure 7:**
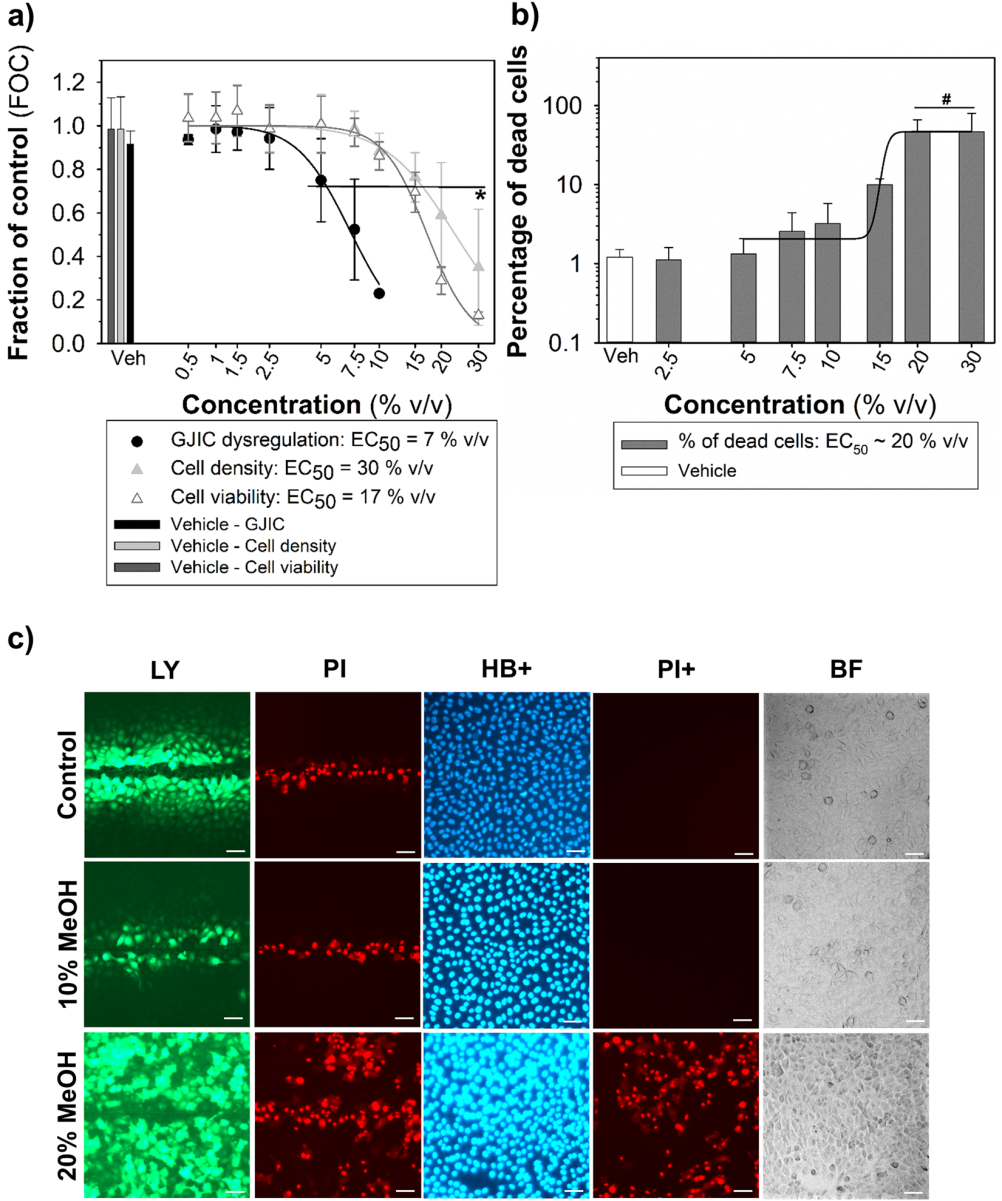
Methanol as a cytotoxic GJIC-inhibitor assessed using the multiparametric SL-DT assay in rat liver progenitor WB-F344 cells (a 24-well plate format, 0.5-h exposure). (**a**) The effects were compared with the non-treated control and expressed as the fraction of control (FOC; FOC_non-treated control_ = 1.0). Means (SD) of independent experiments are presented (n = 3). The “*” denotes significant differences at P ≤ 0.05 (the exact P-values for GJIC: 0 *vs*. 7.5% v/v – 0.010, 0 *vs*. 10% v/v – <0.001; for density: 0 *vs*. 20% v/v – 0.027, 0 *vs*. 30% v/v – <0.001; for viability: 0 *vs*. 15% v/v – 0.001, 0 *vs*. 20 and 30% v/v – <0.001) compared to the vehicle control (one-way ANOVA with Dunnett’s test). (**b**) The % of dead cells were calculated as % of propidium iodide (PI)-stained cells compared to Hoechst 33342 (HB)-stained cells. Means (SD) of independent experiments are presented (n = 3). The “#” denotes significant differences at P ≤ 0.05 compared to the vehicle control (Kruskal-Wallis ANOVA with Dunnett’s test). (**c**) The representative images of communicating cells stained with Lucifer Yellow (LY), the initial loaded cells along the cut stained with propidium iodide (PI), the total cells stained with Hoechst 33342 (HB+) and dead cells stained with PI (PI+). Bar = 50 μm.

Multiparametric assay is easily adaptable for automated image acquisition using a fully motorized fluorescence microscope, such as Axio Observer Z1 equipped with Zen blue 2.3 software. Especially in the case of microplate-format of innovative multiparametric SL-DT assay, large image datasets can be rapidly produced, for example for 24-well plate: 24 wells × 3 cuts per well × 3 channels = 216 images. Manual processing and image analysis of such large image numbers would then become a significant bottleneck for the assay throughput. Therefore, we developed in-house three macros, using open source software Fiji/ImageJ. The macros provide effective (semi)-automated batch detection and quantification of (1) the area of cells communicating through GJIC (Lucifer Yellow); (2) the area of initially dye-loaded cells (propidium iodide along the cut), both by ImageJ Cut_Analyzer Macro; (3) the total number of cells (Hoechst 33342 nuclear staining outside of the cut) by ImageJ Density_Analyzer Macro; and 4) the amount of dead cells (propidium iodide nuclear staining outside of the cut) by ImageJ Viability_Analyzer Macro (Fig. 1). The full-text of all three macros is provided in Supplementary Information as Annex I–III with detailed instructions how to install macros in Supplementary Text S1. Each macro allows unbiased and much faster analysis (0.5–6 sec per image) in comparison to (subjective) manual analysis (3–300 s per image) (Supplementary Table S2). All three macros were successfully used for different formats of cell culture plasticware (Figs. 2 and 3), different cell types (Fig. 4 and Supplementary Fig. S5), and different chemical treatments (Figs. 5–7). The enlarged section of raw and segmented image with Hoechst nuclei staining of WB-F344 cells is shown in the Supplementary Fig. S4 to demonstrate an accuracy of cell counting by Density_Analyzer Macro in confluent and dense cell populations.

**Figure 8:**
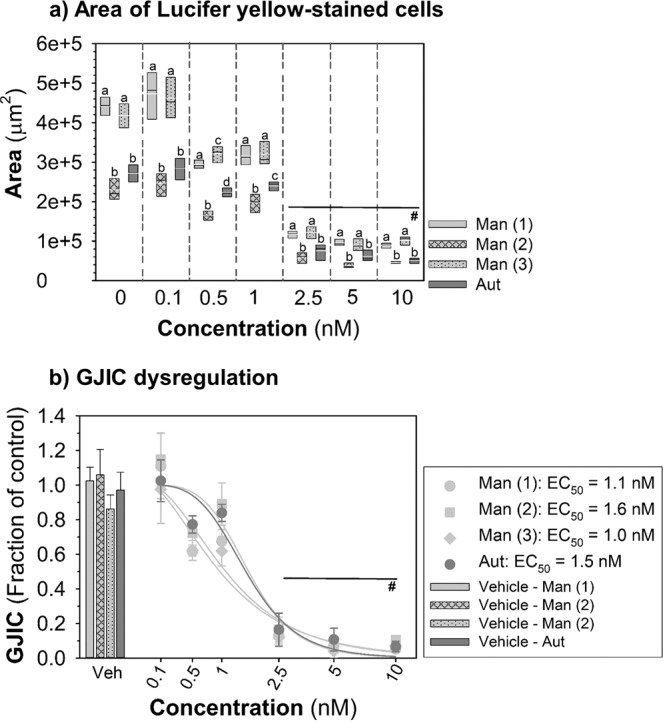
The comparison of manual and (semi)automatic image analyses of GJIC dysregulation caused by TPA in liver progenitor cells WB-F344 (1–h exposure, a 24-well plate format). Three independent evaluators manually analysed GJIC in the same set of pictures (Man (1–3)) and the results were compared with (semi)automatic analysis using the in-house-built macro (Aut). (**a**) Box plots show mean (*white*), median (*black*) and 25–75 percentile of the area of Lucifer Yellow-stained cells (n = 4). The values with different lowercase letters differ significantly among the individual image analysis for the specific concentration (P ≤ 0.05, one-way ANOVA with Tukey’s multiple comparison test). “#” denotes significant differences (P ≤ 0.05, Kruskal-Wallis ANOVA with Dunnett’s test) compared to the vehicle control within each image analysis. (**b**) The inhibition of GJIC plotted as a fraction of control (FOC; FOC_non-treated control_ = 1.0). Means (SD) are presented (n = 4–6). The “#” shows significant differences (P ≤ 0.05, Kruskal-Wallis ANOVA with Dunnett’s test) compared to the vehicle control within each image analysis. TPA, 12-O-tetradecanoylphorbol-13-acetate.

To compare performance of the macro with the manual evaluation, three evaluators assessed manually (using Fiji/ImageJ software) the area of Lucifer Yellow-stained WBF-344 cells after the treatment with TPA. This manual analysis was relatively subjective and differed among the evaluators (Fig. 8a). Two evaluators Man (1) and (3) got similar absolute areas, but those differed from the areas evaluated by the evaluator Man (2). When we compared the results obtained from the manual analyses with the automatic analysis of the same set of the images by the ImageJ Cut_ Analyzer Macro, the macro produced results close to the evaluator Man (2) (Fig. 8a). On the other hand, the response to TPA did not differ either among the manual analyses or between the manual and automatic analyses with the similar EC_50_ values of 1–1.6 nM (Fig. 8b) after data normalization.

### Multiparametric SL-DT assay is suitable for different cell types

**Figure 9:**
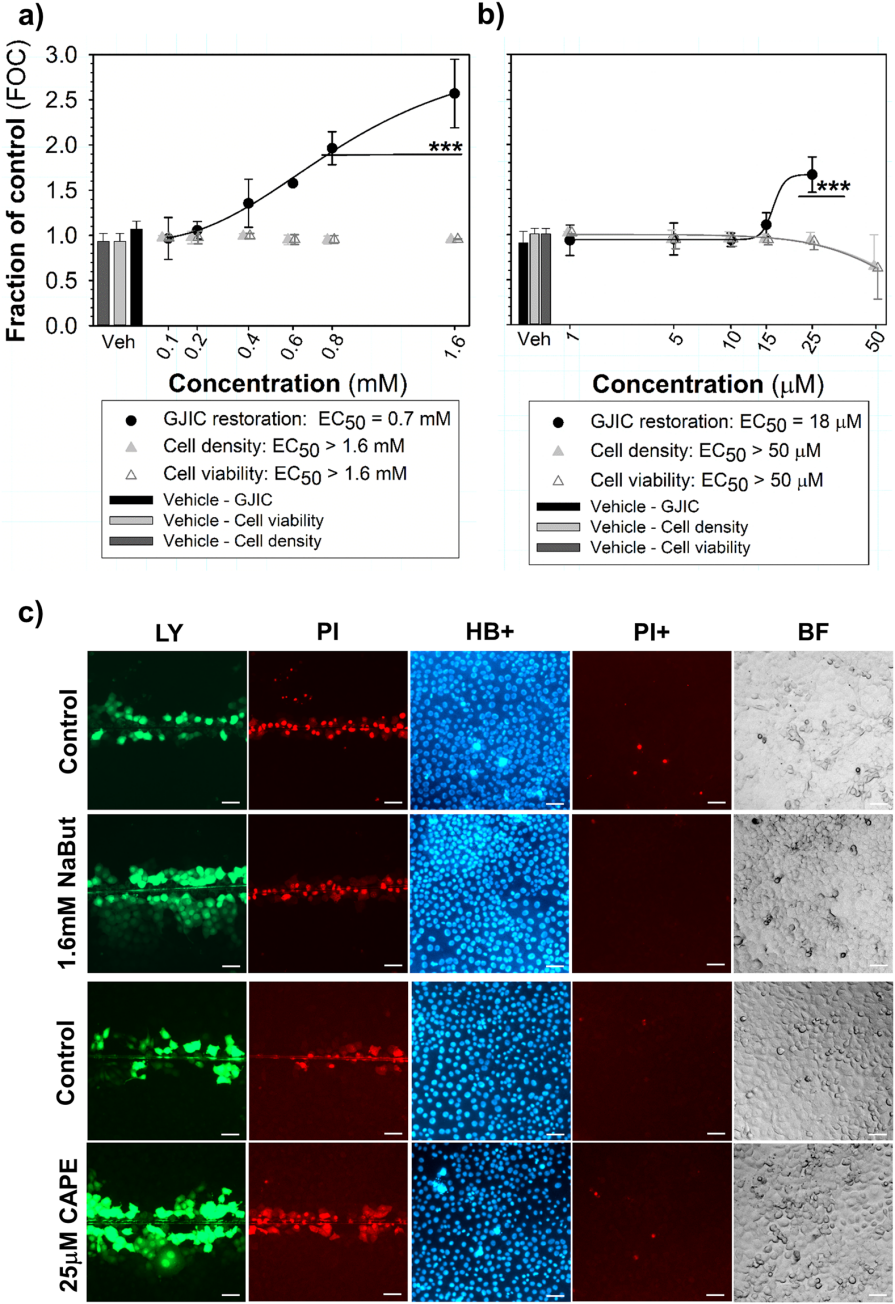
Sodium butyrate (**a**) and caffeic acid phenethyl ester (**b**) as GJIC stimulators assessed using a multiparametric assay in a GJIC-deficient WB-F344-ras cells (*ras*-transformed rat liver progenitor WB-F344 cells, a 24-well plate format, 24-h exposure). (**a**,**b)** The effects were compared with the non-treated control and expressed as the fraction of control (FOC; FOC_non-treated control_ = 1.0). Means (SD) of independent experiments are presented (n = 3). “***” denote significant differences at P ≤ 0.001 (the exact P-values for sodium butyrate: 0 *vs*. 0.8 and 1.6 mM – <0.001; for caffeic acid phenethyl ester: 0 *vs*. 25 μM – <0.001) compared to the vehicle control (one-way ANOVA with Dunnett’s test). (**c**) The representative images of communicating cells stained with Lucifer Yellow (LY), the initial loaded cells along the cut stained with propidium iodide (PI), the total cells stained with Hoechst 33342 (HB+) and dead cells stained with PI (PI+). Bar = 50 μm.

To set up the multiparametric SL-DT assay, WB-F344 cell line was employed as one of the most widely used model for the *in vitro* assessment of GJIC^9,10^. Nevertheless, multiparametric microplate-based SL-DT assay is suitable also for other cell types, as demonstrated in this study. In addition to normal rat liver progenitor WB-F344 cells, we used the assay also for other GJIC-competent cells, such as human respiratory epithelial cells (human bronchial epithelial BEAS-2B and HBE1 cells) and mouse somatic testicular cells (Sertoli TM4 and Leydig TM3 cells) (Fig. 4 and Supplementary Fig. S5), as well as for GJIC-deficient cells, such as WB-F344 cells transfected with H-*ras* oncogene (WB-F344-ras) (Fig. 9). TPA inhibited GJIC in all types of GJIC-competent cells, with the EC_50_ values ranged between 1–7 nM (Supplementary Figs. S6) while causing no effects on density or viability (Fig. 4, Supplementary Figs. S5 and S7). All three macros (ImageJ Cut_, Density_ and Viability_Analyzer Macros) were found to function for most of these cell types and endpoints. The most difficulties were experienced with the macro for total cell (nuclei) counting (ImageJ Density_Analyzer Macro), whose efficiency highly depends on the cell type and the level of the cell density. While functioning well for most of the cell lines used in this study (Supplementary Fig. S4), the macro was not able to effectively segment small, touching or overlapping nuclei in the images of high-cell density and overconfluent cultures of Sertoli TM4 cells, obtained under the microscope settings used in this study (Supplementary Fig. S7). Therefore, these cells were counted manually according to the established protocol (Supplementary Text S2).

### Multiparametric SL-DT assay is well-suited for assessment of chemicals causing a different cellular response

To validate the assay, WB-F344 or WB-F344-ras cells, respectively, were treated with concentration ranges of different types of compounds representing known GJIC blockers, cytotoxic compounds, or GJIC stimulators. Similarly to TPA (Figs. 2 and 3), another model GJIC blocker, 18-β-glycyrrhetinic acid, dysregulated GJIC after 2-h exposure with the EC_50_ value = 39 μM (Fig. 5a). Unlike TPA, 18-β-glycyrrhetinic acid was also found to decrease both cell density and viability at the highest experimental concentrations (EC_50_ values: ~200 μM) (Fig. 5a), and increase the percentage of dead cells (EC_50_ value = 220 μM; Fig. 5b-c). A known GJIC-inhibiting polycyclic aromatic hydrocarbon, fluoranthene, was also assessed in this multiparametric assay in WB-F344 cells, with EC_50_ value = 30 μM (1-h exposure), while not causing cytotoxic effects up to 100 μM (Fig. 6a–c). Due to UV light fluorescence of fluoranthene, relatively higher fluorescent intensity of fluoranthene-treated WB-F344 cells was observed under DAPI channel in comparison to control cells, however, this issue did not affect cell counting by ImageJ Density Analyzer Macro (Fig. 6c). Alcohol methanol also dysregulated GJIC (EC_50_ value = 7%, v/v), but almost coincidentally decreased both cell density (EC_50_ value = 30%, v/v) and fraction of viable cells (EC_50_ value = 17%, v/v) in comparison with control, while simultaneously increasing the percentage of the dead cells in the population (EC_50_ value = 30%, v/v) (Fig. 7a–c).

In addition to GJIC dysregulation, the multiparametric assay also allows the assessment of GJIC induction or restoration. Sodium butyrate restored cell communication in *ras*-transformed and GJIC-defective WB-F344-ras cells (EC_50_ value = 0.7 mM) without any toxic effects up to 1.6 mM (Fig. 9a,c). Caffeic acid phenethyl ester stimulated GJIC in WB-F344-ras cells (EC_50_ value = 18 μM) as well, but simultaneously decreased the relative numbers of total and viable cells (Fig. 9b,c), and significantly increased the share of dead cells in the culture (Supplementary Fig. S8) at the highest tested concentration (50 µM).

## Discussion

Due to key role of GJIC in coordination of signalling events and its involvement in toxicant-responses, various pathophysiological conditions and diseases, it provides an interesting biological endpoint for the assessment of adverse health effects of environmental agents, toxins or pharmaceuticals, as well as for the assessment of promising GJIC-targeting activities during the development of new therapeutics^11,15,17,18^. Currently, there is a wide variety of methods to assess GJIC, e.g. metabolic cooperation, microinjection, radioactive metabolite transfer, electro-coupling or fluorescence recovery after photo-bleaching^19,20^. As summarized in the Supplementary Table S3, some of these methods for GJIC assessment have been recently adapted for HTS and/or HCA/HCS. These assays are mostly based on commonly used dye-transfer techniques such as electroporation^21^, laser perforation^22^, microinjection^23^, microfluidic loading^24,25^, parachute/preloading assay^26–28^, or on a luminometric or fluorimetric sensing of specific molecules exchanged via gap junctions between donor and recipient cells^29,30^. However, these methods can require a special equipment (electroporating^21^, microscopic^22,27,28^, microinjecting^23^, microfludic^24,25^), that is not commonly available in a laboratory, and proprietary, closed source software for high-throughput image analysis^21,22,26,28^, or rely on specialized transduced cell models^29,30^.

Similarly to multiparametric SL-DT assay described in this study, parachute or preloading assays also do not need any specialized equipment, materials or cell lines. In contrast to SL-DT, integrity of the cells is maintained in both parachute or preloading assays, since the cells are non-invasively loaded with a membrane-permeable tracer, such as methyl ester-fluorescent dyes, e.g. calcein-AM. After removal of methyl ester group by intracellular esterases in the loaded cells, a gap junction permeable and membrane-impermeable calcein is formed. Preloading method is based on formation of a confluent monolayer from a mix of suspended loaded and unloaded cells^31^, whereas resuspended loaded cells are let to adhere onto a monolayer of unloaded cells in the parachute assay^32^. Within the both assays, the spread of the dye from donor to recipient cells can be monitored with an epifluorescence microscope. Both techniques represent a very good platform for HTS with a high level of automation and few manual steps^26–28^. However, as any other method for monitoring GJIC, also these techniques possess some disadvantages (Supplementary Table S3), including: (1) need for the trypsinization of the donor cells, which itself might be considered as a relatively invasive step, (2) the need for formation of gap junction channels between donor and recipient cells during the chemical exposures and limited opportunities to study rapid mechanisms of GJIC inhibition via gating of gap junction channels), (3) a requirement of a relatively long time lag for the reattachment of cells (15 min-5 h), (4) the ability of the cells to form gap junctions in these assays depends enormously on the cell type, (5) gap junction-nonspecific dye transfer of calcein has been observed, (6) and calcein can be actively pumped out from some cell types by multidrug resistance proteins^19,33^. Thus, alternative methods for HTS and/or HCA/HCS assessment of GJIC might be still useful and needed to suit broader research needs.

Interestingly, none of GJIC assays recently adapted for HTS and/or HCA/HCS is based on the traditional SL-DT assay introduced by El-Fouly *et al*. in 1987^16^. SL-DT is a simple, convenient, and relatively uncomplicated method with minimum requirements for specialized, usually expensive, equipment/supplies, allowing for assessing functional gap junction coupling within *in vitro* cell populations^16,17^. Therefore, SL-DT has become one of the most commonly used techniques for identifying and mapping GJIC in a wide variety of cell lines^19^. However, the protocols for SL-DT were not modified much since 1987^14,15^. The main disadvantage of this original method is its relatively low throughput, given particularly by the traditional 35 mm Petri dish design. Moreover, it has been demonstrated that digital images of SL-DT can be processed and quantified by image analysis software^20^, but there has been a lack of available, ideally open source tools, optimized for a (semi-)automated batch segmentation and quantification of SL-DT experiments. These factors altogether might have represented significant limitations and bottlenecks for SL-DT method to be considered for HTS/HCA toxicity screening programs, such as EPA’s Toxicity Forecaster (ToxCast), Tox21 assays, or for other HTS/HCA studies screening for *in vitro* effects of toxicants, pharmaceuticals or other bioactive compounds. Here, we introduced the innovative multiparametric SL-DT assay allowing the assessment of GJIC along with evaluation of cell density and viability, which can be conducted without a need of specialized equipment, consumables, technical skills, complex setups and protocols.

In addition to Lucifer Yellow as a dye passing through gap junctions, we incorporated two other fluorophores into the dye loading step of the innovative SL-DT assay: propidium iodide as an intact cell membrane impermeable dye, and Hoechst 33342, as a cell membrane permeable dye. Propidium iodide substitutes a lysine-fixable, fluorescent dextran such as Rhodamine-, Texas Red- (used in this study) or TRITC-labelled dextran^14,19^. Propidium iodide is positively charged (2+) low molecular dye (MW of the fluorescent ion: 414), which is unable to cross intact cell membranes^19^. Despite its low molecular weight, small enough to pass through gap junction channels, propidium iodide has limited diffusion through gap junction channels due to strong binding to DNA^34,35^, as observed also in our study. The propidium iodide-staining was primarily limited to a single cell row close to the cut, and no substantial amount of dye passing to the second cell row was detected, very similarly to much larger molecule, Texas Red-dextran.

As demonstrated by this study (Supplementary Table S1), propidium iodide can be used to label the loaded cells at working concentrations 500-times lower than nearly three times more expensive labelled dextran. Similarly to Lucifer Yellow, propidium iodide was also found to be relatively more stable in solution than labelled dextrans. The replacement of labelled dextrans with propidium iodide thus allows to re-use Lucifer Yellow dye-loading solution up to 10-times or more, without major issues with a signal fading. This significantly reduces the costs of the assay in comparison with the traditional setup.

Moreover, propidium iodide is commonly used, in fluorescence microscopy and flow cytometry as a marker of dead cells with compromised membrane integrity^36,37^. Thus, the use of propidium iodide in the multiparametric SL-DT assay allows for image-based quantitation of this classical marker of dead cells outside of the cut region. To account for more severe cytotoxic effects leading to the cell detachment and loss, or changes in cell density due to inhibited or increased cell proliferation rate (particularly during longer exposures and treatments), Hoechst 33342 was added into our innovative SL-DT method to stain nuclei of all cells. In addition to providing information about a total cell count and changes in cell density, this parameter in combination with the number of dead cells allows quantifying number of viable cells and % of dead cells in the culture. This feature of the innovative SL-DT assay thus minimizes the needs for conducting parallel cytotoxicity/viability assay during screening for GJIC effects. For example, 18-β-glycyrrhetinic acid was found to significantly inhibit GJIC at concentrations ≥25 μM, but cytotoxicity became a dominant factor at concentrations >150 μM, as documented by the decrease in total cell density, decrease of numbers of viable cells, and increased percentage of dead cells. This allows to identify GJIC effects occurring at non-cytotoxic concentrations of gap junction regulators (blockers or stimulators), and discriminate them from GJIC alterations associated with decreased cell viability and/or reduced cell density, when the levels of GJIC can reduce due to loss of physical contact between the neighbouring cells. It improves interpretation of the results from SL-DT assay with respect to chemical hazard identification and assessment, when inhibition of GJIC with effective concentrations well below cytotoxicity can have different toxicological relevance than compounds affecting GJIC at subcytotoxic levels. In drug and bioactive compound screening, chemicals and samples altering GJIC only at cytotoxic concentrations might not be of interest for the treatment of diseases, such as ischemia or cancer. Finally, Hoechst 33342 and propidium iodide staining to quantify total and dead cells should be compatible also with other HCA/HCS methods used for GJIC assessment, as demonstrated for parachute assay and evaluation effects of cigarette smoke^26^.

Besides being a component of gap junctions among adjacent cells, connexin molecules can form functional undocked connexons in nonjunctional membranes, known as connexin hemichannels^38^. Other proteins called pannexins, a family of chordate proteins homologous to the invertebrate innexins, are known to form channels in the plasma membrane which are connecting intracellular and extracellular space in mammalian cells. Connexin hemichannels and pannexin channels thus have a role that is distinct from that of GJIC by allowing exchanges small molecules with the extracellular space^39^. Hemichannels remain preferentially closed under normal physiological conditions, mainly due to ambient levels of Ca^2+^. Their opening is regulated by various physiological and pathological conditions, offering a diffusional transmembrane route between the intra and extracellular milieus^40^. Although opening of connexin hemichannels/pannexin channels can be evaluated also by the use of hemichannel-permeable fluorescent dyes such as Lucifer Yellow^41^, in case of such hemichannel assay, the cells are exposed to fluorescent tracers such as Lucifer Yellow or propidium iodide in Ca^2+^/Mg^2+^-free buffer from the extracellular space, while the dye is not introduced into the cells by cut or any other method. Then, the extracellular dye is washed-out with buffer containing Ca^2+^/Mg^2+^ to shut the hemichannels, or the washed cells are quickly fixed using an aldehyde. The cells retaining the tracer are visualized and counted by the fluorescence microscope and appropriate software. Since hemichannel assay depends on hemichannel-mediated dye uptake from the extracellular space, and not on the intercellular dye transfer as for GJIC assessment, both processes can be discriminated. Opening of hemichannels during the SL-DT assay would lead to increased Lucifer Yellow staining of the cells uniformly across the entire cell culture/FOV, not only in the proximity of the dye-loading cut. Moreover, in our SL/DT assay protocol, the cells are washed with CaMg-PBS prior the loading of dyes dissolved also in CaMg-PBS, which should inhibit hemichannel activity. It has been demonstrated that the same *in vitro* model can be used for evaluation of both hemichannel activity by dye uptake assay, and GJIC by SL-DT assay^42–44^. Interestingly, some chemicals can regulate hemichannels and gap junction channels differently. For example, proinflammatory cytokines from activated microglia opened Cx43 hemichannels, but inhibited GJIC in astrocytes^45^. On the other hand, the Cx43 mimetic peptide Gap19 inhibited hemichannels without altering gap junctional communication in astrocytes^46^. Altogether, hemichannel activity likely did not interfere with the GJIC assessment by multiparametric SL-DT assay.

The multiparametric GJIC assay was found to work in miniaturized microplate formats without changing cell responses to chemicals. In contrast to traditionally used 35-mm Petri dishes, a multiwell microplate format increases SL-DT assay throughput (due to easier plate handling, possibility to use multichannel pipettes etc.) and enables rapid testing of large amount of chemicals in a short time even without robotic automation of the workflow. However, the microplate format of SL-DT assay allows to use HTS setups with automatic cell seeding, washing or dispensing/exposing steps, with the exception of relatively simple and quick (<5 min per plate) manual dye-loading step, which has yet to be automated (i.e. by integration of robotic cutters). The microplate format facilitates also the speed of image acquisition even with the use of manual (non-motorized) microscopes, due to easier plate handling, faster navigation between different replicates and experimental treatments, and faster localization of the cut in smaller cell growth areas. The speed of image acquisition can be significantly increased by the use of automated microscopes or HCA/HCS readers with motorized stages, automatic focusing and image acquisition workflow, possibly equipped also with microplate handling and loading^47^.

Multiparametric microplate-based SL-DT assay conducted with a higher throughput can generate large sets of microphotographs, which need to be effectively and rapidly analysed. Thus, we have developed in-house semi-automated macros for batch processing and analysis of images from SL-DT. As traditional manual cell counting is time-consuming and less accurate method prone to evaluator bias and errors^19^, our macros provide more objective, faster and precise approach to get reliable results. The evaluation of 216 images from a 24-well plate (24 wells × 3 cuts × 3 channels) can be accomplished within minutes compared to hours of manual analysis. Our image analysis tools were developed within an open source software package Fiji/ImageJ and they are publicly available to potential future users, which represents another benefit in contrast to other existing semi−/automated tools for image-based GJIC analysis, which are usually based on Matlab^22,48,49^ or other proprietary, closed source software^26,28^.

Importantly, we demonstrated applicability of our innovative multiparametric SL-DT assay for variety of *in vitro* cell types such as liver cells (WB-F344), lung cells (BEAS-2B, HBE1) or testicular cells (TM4 and TM3 cells), which all are GJIC-competent cells commonly used for the assessment of GJIC in response to different chemicals^50–54^ or physical treatments^55^. All these cell types express connexin 43^51,56,57^, which is widely expressed in many cell types (especially in the epithelial cells) and whose gap junction channels are diffusible for Lucifer Yellow fluorescent dye^58^. In addition to gap junctions formed by connexin 43, Lucifer Yellow has been reported to transfer through channels formed by a number of other connexin types, such as connexin 26, 31, 32, 37, 40, 45, 46, 47^34,58–62^. However, the transfer of Lucifer Yellow through the gap junction channels built from some other connexin isoforms (e.g. connexin 30, 36 and 57, and mouse connexin 30.2 and its human ortholog 31.9^58,63–66^) might be limited. In that case, other gap junction-permeable fluorescent trackers excitable at ~488 nm shall be used (reviewed in Abbaci *et al*.^19^). As demonstrated in this study, dyes such as calcein or CF™ 488A can be easily incorporated as an alternative to Lucifer Yellow into the set-up of this assay.

Our innovative SL-DT assay can be used for screening of GJIC blockers and stimulators within toxicity assessment of environmental or food toxicants, drugs, as well as during development of new therapeutics targeting GJIC and connexins. We verified applicability of this approach by testing known GJIC blockers as well as stimulators. The obtained EC_50_ values for TPA in studied cell lines were comparable to range of the values published in previous studies^51,67^. Similarly, GJIC-inhibitory activities of fluoranthene and 18-β-glycyrrhetinic acid in WB-F344 cells evaluated by our innovative assay were similar with previously reported studies^26,52,68^. By screening GJIC stimulators, we confirmed that our multiparametric assay can be also used for assessment of chemopreventive agents, such as caffeic acid phenethyl ester^69^ or sodium butyrate^70^, which were previously found to restore cell-cell communication in neoplastically transformed, GJIC-deficient WB-F344-ras cells. This assay can be used in pharmaceutical or biomedical research to evaluate pharmaceutically-relevant chemicals, bioactive compounds, extracts, or therapeutic treatments, for their ability to alter GJIC in desired cell types (e.g. malignant cells) or under experimentally induced chemical stress, diseased-conditions (e.g. tumor promoting, inflammatory)^14,15,71,72^.

In conclusion, we developed an innovative SL-DT assay for medium- or high-throughput analyses of GJIC as well as primary screening for the identification of potential gap junction blockers and stimulators. This assay is easy to perform, time and cost efficient, and reliable method applicable for a variety of cell types. In comparison to other HTS/HCA assays for GJIC assessment, there are no additional steps during the experiments, such as cell transduction, preloading with tracers, trypsinisation, mixing of donor and acceptor cells, cell stimulation. The innovative assay requires only (1) a cell culture of the interest, grown in its respective cell culture medium; (2) common cell culture plasticware, preferably a microplate format with 24- to 96-wells; (3) micro knife with a blade fitting the dimensions of respective microplate wells; (4) common fluorophores (Hoechst 33342, Lucifer Yellow, propidium iodide), whose working solution can be repeatedly used; (5) common buffers and fixatives (PBS, formaldehyde); (6) widefield epifluorescence microscope with DAPI, GFP and Texas Red excitation/emission filters; (7) open source macros for Fiji/ImageJ software. The experimental protocol is very straightforward, including following steps: (1) cell seeding and growth prior the exposure, (2) exposure/treatment for desired duration (tens of the minutes up to several days), (3) SL-DT assay, which takes less than 30 min per microplate, and (4) microscopic imaging and analysis.

In addition to its simplicity, low costs and versatility, our multiparametric SL-DT assay also provides further information about cellular responses beyond GJIC assessment, which allows for more insights into effects of compounds, thus enabling us to eliminate compounds acting through unrelated pathways such as non-specific cytotoxic effects. Moreover, this multiparametric assay is an open platform for further development, including evaluation of other cellular endpoints, such as discrimination between apoptosis and necrosis or cell cycle assessment, which can be derived from the dead cell staining and/or the pattern of nuclei staining^73^. Thus, *in vitro* assessment of GJIC using the innovative SL-DT method in the modified microplate version is adaptable for the needs of HTS and HCA, and represents a perspective technique, which could be utilized for multiparametric *in vitro* screening and simultaneous assessment of chemical effects on intercellular communication, cell proliferation, apoptosis and cytotoxicity.

## Materials and Methods

### Chemicals

TPA (purity: ≥99%; Cat. No. P8139; Sigma Aldrich, Prague, Czech Republic), fluoranthene (purity: 98%; Cat. No. F807, Sigma Aldrich), 18-β-glycyrrhetinic acid (purity: 97%; Cat. No. G10105, Sigma Aldrich), methanol (purity: ≥98,5%; Cat. No. 20903, VWR International, Stříbrná Skalice, Czech Republic), sodium butyrate (purity: ≥98,5%, Cat. No. B5887, Sigma Aldrich), caffeic acid phenethyl ester (purity: ≥97%; Cat. No. C8221, Sigma Aldrich). Other chemicals and reagents were purchased from Sigma Aldrich unless noted otherwise.

### Cell lines

All cells were routinely cultured in 25 or 75 cm^2^ tissue culture flasks (TPP, Trasadigen, Switzerland) in humidified 5% CO_2_ atmosphere at 37 °C, and routinely passaged by trypsinisation twice or thrice per week.

### BEAS-2B cell line

Non-differentiated human bronchial epithelial cell line immortalized with Human Adenovirus 12-SV40^74^ was purchased from American Type Culture Collection^®^ (Manassas, VA). BEAS-2B cells were cultured in LHC-9 Gibco medium (Cat. No. 12680013, ThermoFisher, Waltham, MA) without any supplements (pH: 7.40). For the experiments, BEAS-2B cells were seeded in a density of 50,000 cells/cm^2^ (0.5 mL per well) into a 24-well plate pre-coated with a mixture of fibronectin (0.01 mg/mL; Sigma Aldrich, Cat. No. F1141), bovine collagen type I (0.03 mg/mL; cat. no. 125-5, Cell Applications, San Diego, CA), and bovine serum albumin (1 mg/mL; Cat. No. A9085, Sigma Aldrich). 48 h after cell seeding, the growth medium was exchanged with the exposure medium (culture medium with a diluted toxicant; TPA) and the cells were exposed for 3 h.

### HBE1 cell line

Non-tumor, non-cystic fibrosis human bronchial epithelial cell line immortalized with Human Papilloma Virus-18 E6 and E7 oncogenes^75^ was generously provided by Dr. A. K. Bauer (University of Colorado, USA). HBE1 cells retain ion transport and other properties comparable to the native cells^75^. HBE1 cells were cultured in DMEM/F12 (Cat. No. L0094, Biowest, Nuaillé, France) medium supplemented with 4 µg/mL human insulin, transferrin (5 µg/mL), human epidermal growth factor (10 ng/mL), dexamethasone (0.1 µM), cholera toxin (20 ng/mL), Plasmocin^TM^ (2.5 µg/mL, Invivogen, San Diego, CA), L-glutamine (2.5 mM), endothelial cell growth supplement (0.1% v/v, Promocell, Heidelberg, Germany) (pH: 7.40). For the experiments, HBE1 cells were plated in a density of 50,000 cells/cm^2^. into a 24 well-plate (0.5 mL/well). 48 hrs after cell seeding, the growth medium was exchanged with the exposure medium (culture medium with a diluted toxicant; TPA) and the cells were exposed for 24 h.

### TM3 and TM4 cell lines

Leydig TM3 and Sertoli TM4 cells, non-tumorigenic cell lines derived from mouse testis, were purchased from the American Type Culture Collection. These somatic testicular cells share many characteristics of pre-pubertal Leydig or Sertoli cells *in situ*, respectively^76,77^. Both cell lines (passages <25) were grown in DMEM/F12 (Cat. No. D6434, Sigma Aldrich), supplemented with horse serum (5% v/v; PAA, Prague, Czech Republic), foetal bovine serum (2.5% v/v), and L-glutamine (2.5 mM) (pH: 7.40). For the experiments, Leydig TM3 cells or Sertoli TM4 cells were seeded in a density of 26,000 or 30,000 cells/cm^2^ into a 24 well-plate pre-coated with gelatine (0.1%, w/v). 48 hrs after cell seeding, the growth medium was exchanged with the exposure medium (culture medium with a diluted toxicant; TPA) and the cells were exposed for 1 h.

### WB-F344 cell line

Rat liver epithelial cell line isolated by Drs. J.W. Grisham and M.S. Tsao of the University of North Carolina^78^ was generously provided by Prof. J. Trosko (Michigan State University, USA). WB-F344 cells are non-tumorigenic and possess characteristics of rat liver oval/progenitor cells^79^. WB-F344 cells were grown in DMEM Gibco medium (Cat. No. 11880, ThermoFisher) supplemented with foetal bovine serum (7% v/v) and L-glutamine (2 mM) (pH: 7.40). For the experiments, the cells were seeded in a density of 25,000 cells/cm^2^ in 35-mm Petri dish (2 mL/dish), a 24-, 48- or 96-well plate (0.5 mL, 0.5 mL or 0.25 mL/well). 48 h after cell seeding, the growth medium was exchanged with the exposure medium (culture medium with a diluted toxicant: TPA, fluoranthene, 18-β-glycyrrhetinic acid, methanol) and the cells were exposed for 0.5 h (methanol), 1 h (TPA, fluoranthene) or 2 h (18-β-glycyrrhetinic acid).

### WB-F344-ras cell line

WB-F344-ras cells were derived by transducing WB-F344 cells with retroviral vector containing v-Ha-*ras* oncogene^80,81^. The cells were generously provided by Prof. J. Trosko (Michigan State University, USA). WB-F344-ras cells are tumorigenic, with numerous characteristics of neoplastically transformed cells, including aberrant connexin localization and reduced levels of GJIC^80,81^. WB-F344-ras cells were cultured in DMEM Gibco (Cat. No. 11880, ThermoFisher) supplemented with foetal bovine serum (7% v/v) and L-glutamine (2 mM) (pH: 7.40). For the experiments, the cells were seeded in a density of 20,000 cells/cm^2^ into a 24 well-plate. 48 h after seeding, the growth medium was exchanged with the exposure medium (culture medium with a diluted compound: sodium butyrate or caffeic acid phenethyl ester) and the cells were exposed for 24 h.

### Traditional SL-DT

The traditional SL-DT technique^16^ was done according to recently published protocols^14,15^. This method allows to successfully load Lucifer Yellow into the cells by a cut with a micro knife blade, presumably as a result of mechanical perturbation of the membrane, in a reproducible manner which minimizes the area of loaded cells and is not leading to an extensive cell damage or detachment. Lucifer Yellow becomes incorporated by the cells along the cut. As normal membrane permeability is re-established (seconds), Lucifer Yellow becomes trapped within the cytoplasm and move from the dye-loaded cells into adjacent ones connected by functional gap junction channels. For the verification that the dye transfer occurs through intercellular junctions, the high molecular weight, plasma membrane-impermeable and gap junction-impermeable fluorophore, such as Texas Red-dextran, serves to label dye-loaded cells along the cut. Only the dye-loaded cells reveal red fluorescence, whereas the dye-loaded cells as well as a number of adjacent cells reveal green fluorescent colour in cultures of GJIC-competent cells (Fig. 1d). Briefly, the exposed cells were rinsed with calcium- and magnesium-supplemented PBS (CaMg-PBS; pH 7.2). The solution of Lucifer Yellow CH dilithium salt (1 mg/mL) and Texas Red-dextran (5 mg/mL; 10 kDa, ThermoFisher) in CaMg-PBS was added to the cells and the dyes were introduced to the cells by parallel cuts (35-mm Petri dish: 3 cuts per dish) with a micro knife blade (Pro Stencil Knife Blade #17, Proedge Blades, Paterson, NJ). After 10 min, the cells were washed with CaMg-PBS to remove background fluorescence and fixed in 4% formaldehyde in PBS. Lucifer Yellow dye in solution is stable, thus the staining solution can be collected and reused at least 10-times, when stored in dark and refrigerated. However, signal from Texas Red-dextran fades, when the solution is reused.

### Innovative multiparametric SL-DT assay

The exposed cells were rinsed with CaMg-PBS. The solution of Lucifer Yellow (charge: 2−/MW of the fluorescent ion: 443) CH dilithium salt (1 mg/mL), propidium iodide (charge: 2+/MW of the fluorescent ion: 415) (10 μg/mL), and Hoechst 33342 (charge: 3+/MW: 562) (50 μg/mL) in CaMg-PBS was added to the cells. In some experiments, other fixable polar tracers excitable at ~488 nm were evaluated as alternatives to Lucifer Yellow, also with working concentrations 1 mg/mL in CaMg-PBS: calcein (charge: 4−/MW of the fluorescent ion: 623), FITC (charge: 2−/MW of the fluorescent ion: 389), CF™ 488A (minimal charge compared to Alexa 488 (−1) according to the supplier Sigma Aldrich/MW: 914). After dye addition to the rinsed cells, parallel cuts (35-mm Petri dish: 3 cuts per dish; 24-well plate: 3 cuts per well; 48-well plate: 2 cuts per well; 96-well plate: 1 cut per well) were done with a micro knife equipped with a straight edge stainless steel blade (Pro Stencil Knife Blade #17 for 35-mm Petri dish, 24- and 48-well plates, and Pro Small Chisel Blade #66 for 96-well plate, Proedge Blades; Fig. 1b–c). The areas for cell loading were randomly selected in the central part of the dish or microplate wells, whereas peripheral regions were omitted due to distortions at the edges of culture plates that do not allow good cell growth or optical detection as previously recommended^14,15^. After 10-min incubation, the cells were washed with CaMg-PBS to remove background fluorescence and fixed in 4% formaldehyde in PBS. Dye solution with Lucifer Yellow, propidium iodide and Hoechst can be collected and reused, since it is applied to thoroughly rinsed cells and does not come to the contact with the exposure medium. We reused the dye solution for approximately 10 experiments, when stored in dark and refrigerated. The solution can be filtered through 0.22 μm syringe filter, if needed (e.g. due to accumulation of the cell debris).

### Image acquisition & analysis

The pictures of freshly fixed cells were acquired within 2 h from the end of the treatment. Image(s) from the middle part of each cut (showing the most uniform dye transfer) were captured with a 10× objective (A-Plan 10×/0.25) using a fully motorized Axio Observer Z1 widefield fluorescence microscope (Carl Zeiss, Jena, Germany) equipped with AxioCam 503 Mono camera, Filter Set 38HE (AF 488 channel: excitation - BP 450-490, beamsplitter – FT 495, emission - 525/50) for Lucifer Yellow, Filter Set 20 (Rhodamine channel: excitation - BP 546/12, beamsplitter – FT 580, emission - BP 575-640) for Texas Red dextran and propidium iodide, Filter Set 49 (DAPI channel: excitation – G 365 nm, beamsplitter – FT 395, emission – BP 445/50 nm) for Hoechst 33342, and ZEN blue 2.3 software. The constant exposures were used for all images for a given experiment (Lucifer Yellow: 100–500 ms; Texas Red and propidium iodide: 1000–2500 ms; Hoechst: 100–500 ms). A FOV of 1936 × 1460 pixels corresponding to 1396 × 1053 μm was captured and this image was analysed. The settings for gain, brightness and contrast were same for all images within a set of experiments. If there was editing of the images (brightness or contrast) prior to quantification, all images within a given experiment/set of images were equally edited. For image presentation, each presented picture was cropped to 602 × 602 pixels (except uncropped tiled images in the Supplementary Fig. S2 and S3).

Image analysis was performed with in-house made macros for multiparametric SL-DT assay (Supplement Text S1) created in Fiji, which is a distribution of open source NIH ImageJ software (http://imagej.nih.gov/ij/)^82^. The Cut_Analyzer provides detection and quantification of the area of cells communicating through GJIC (the cells stained with Lucifer Yellow) as well as area of the dye-loaded cells along the cut (the cells stained with propidium iodide). The net area of dye transfer was obtained by subtracting propidium iodide area from the Lucifer Yellow area for each image. The Density_Analyzer provides the total number of cells (cells stained with Hoechst in the predefined area outside of the cut), while the Viability_Analyzer provides the number of dead cells (propidium iodide-stained cells in the predefined area outside of the cut). The number of viable cells was calculated by subtracting the number of the dead cells from the number of total cells. The results are saved automatically in .xls format. Within this whole study, all three macros were successfully used for different formats of cell culture plastics and cell types unless stated otherwise. Since some cell types with small and/or overlapping nucleuses such as TM4 cells are more difficult to quantify, we provide an alternative solution (Supplement Text S2).

### Data and statistics analysis

Appropriate vehicle controls were run at all times, and they did not significantly differ from the non-treated cells. Each experiment was repeated at least three times independently. Data from independently repeated experiments were combined and used for all statistical analyses and comparisons. Quantitative data from all endpoints, i.e. (1) net area of dye transfer for GJIC, (2) total cell number for cell density, and (3) number of viable cells only for cell viability, derived from each image were compared with the corresponding values obtained from non-treated (negative) control from the same experiment, and reported as the fraction of control (FOC-GJIC, FOC-Cell Density, FOC-Cell viability). In addition, percentage of dead cells out of the total cell number was also calculated for each treatment and reported without normalization to the negative control (% of dead cells). One-way ANOVA followed by Dunnett’s or Tukey’s post-hoc tests or two-tailed t-test were used for comparing data passing normality (Shapiro-Wilk’s test) and equal variance tests, whereas Kruskal-Wallis ANOVA on ranks followed by Dunnett’s test or Mann–Whitney U test were used for comparing data with unequal variances and/or non-normal distribution. P-values equal or lower than 0.05 were considered as statistically significant. All statistical analyses were done in SigmaPlot (Systat Software, San Jose, CA). Curve-fitting was done using non-linear, 4-parameter sigmoidal regression models using GraphPad Prism 5 (GraphPad Software, La Jolla, CA). The EC_50_ value was determined for each independent experiment, then the geometric mean of EC_50_ values from the independent experiments was calculated with 95% confidence interval (CI).

## Supplementary information

Supplementary Information.

## Data Availability

All data generated or analysed during this study are included in this published article (and its Supplementary Information files).
