# Supplementary material for: Improved multiparametric scrape loading-dye transfer assay for a simultaneous high-throughput analysis of gap junctional intercellular communication, cell density and viability
Source: Sci Rep. 2020 Jan 20;10:730. doi: 10.1038/s41598-020-57536-3 (PMC6971000; doi:10.1038/s41598-020-57536-3)
Supplement: Supplementary file 1 — Supplementary Information. [file 41598_2020_57536_MOESM1_ESM.pdf]

SUPPLEMENTARY INFORMATION

Improved multiparametric scrape loading-dye transfer assay for a simultaneous high-throughput analysis of gap junctional intercellular communication, cell density and viability

Aneta Dydowiczová, Ondřej Brózman, Pavel Babica, Iva Sovadinová*

Masaryk University, Faculty of Science, RECETOX, Kamenice 5, CZ-62500 Brno, Czech Republic

*sovadinova@recetox.muni.cz

SUPPLEMENTARY TEXT

Supplementary Text S1. Macro Installation

Three semi-automated macros were built: 1) **Cut_Analyzer.ijm** for analysis the areas of Lucifer Yellow-stained cells and dye-loaded cells along the cut (Appendix I), 2) **Density_Analyzer.ijm** for analysis of total number of cells (Appendix II), and 3) **Viability_Analyzer.ijm** for analysis of dead cells (Apendix III). Requirement: Images in .jpg format. How to install and use macros, we summarized in a short video (Apendix IV).

Step-by-step instruction:

1. Download and install FIJI from http://imagej.net/Fiji

2. Copy macro from Appendix I, II or III to "Untitled.ijm" window.

Open Fiji -> *File* -> *New* -> *Text window*

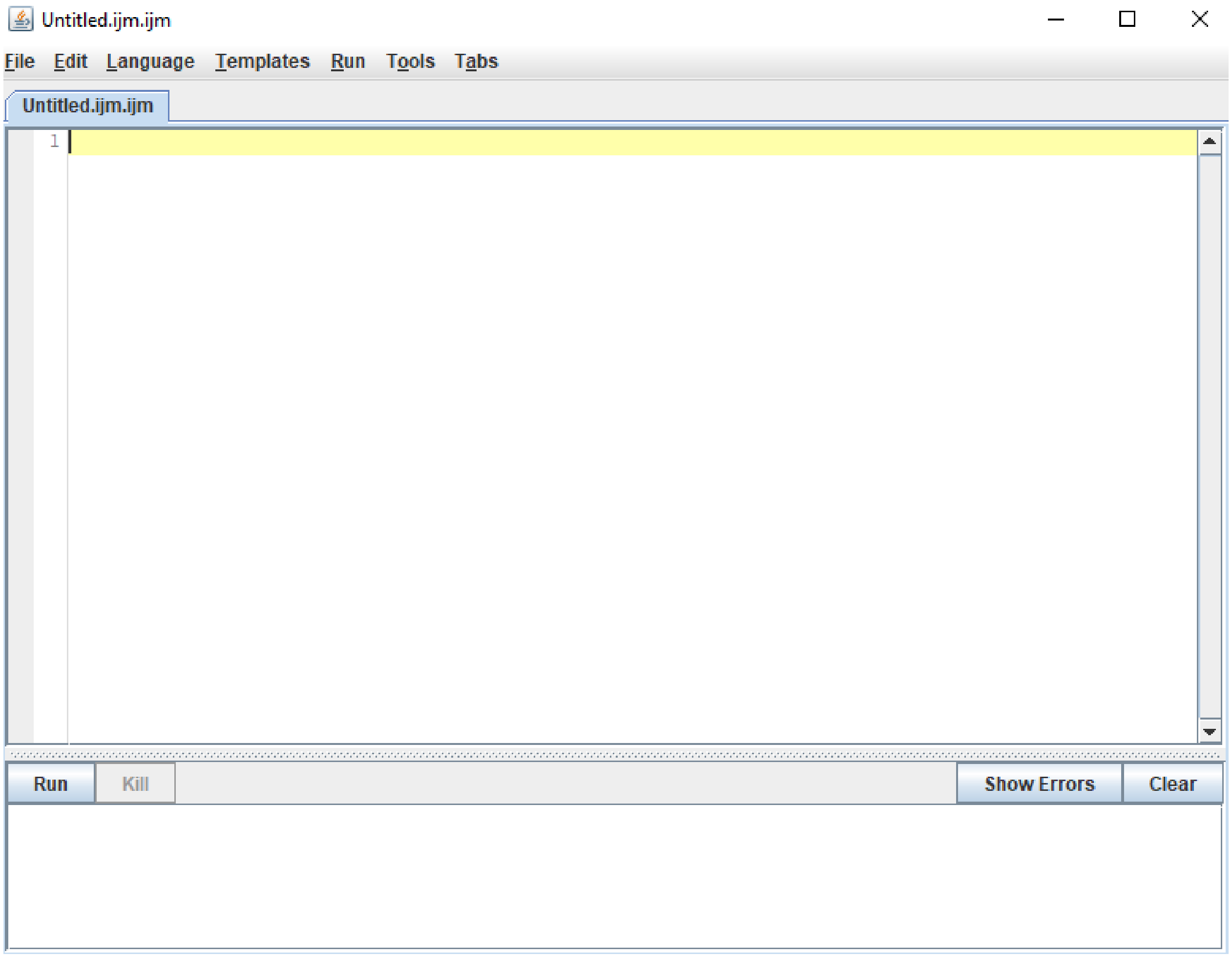

File -> *Save as* -> Cut_Analyzer.ijm or Density_Analyzer.ijm or Viability_Analyzer.ijm-> *Save* -> *Run*

After the installation, the macro are saved in the computer: Open Fiji -> *Plugins*-> *Macros*-> *Run*-> select proper ".ijm" macro

3. Dialog menu is open:

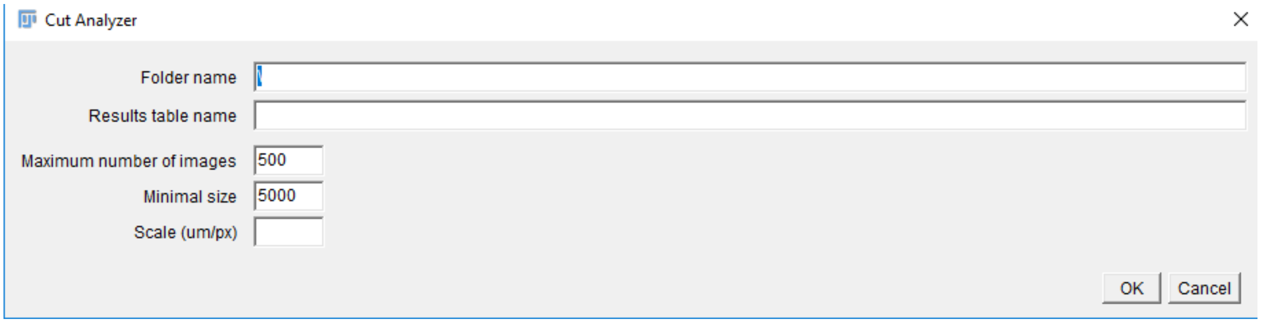

Folder name - Insert pathway to folder with images to be analysed; pay attention to backslash which has to be on the end

Results table name - Write a name of the excel output

Maximum number of images - Insert maximum number of images to be analysed; caution: this depends on capacity of your computer; in case that your folder is too big-> divide images to several folders; default value is set to 500

Minimal size - Set a minimal size of particles to be analysed; for the detection of the area of communicating cells start with the minimal size of 5,000; for detection of loaded cells 3,000 and for cell density or viability 10; all of these values are suitable for scale 0.72 µm/px (images captured at 10× objective and 1× optovar by Zeiss Axio Observer Z1 microscope); approximate minimal size can be assessed by: *open image*-> *analyse* -> *set scale* -> insert px and µm before selecting in toolbar shape of "heart" and outline particle-> *analyse*-> *measure*

Scale - Insert resolution in µm/px

4. Analysis

When analysis is running several windows are open:

1. Toolbar
2. Original image which is analysing
3. Image with detected area of cells or particles
4. ROI Manager - list of all detected particles with possibility to add or delete some
5. Results
6. Summary

7. Log window - shows changes in number of particles; if you are satisfied with analysis close this window; caution: Close only this window. If you close another window, analysis will stop.

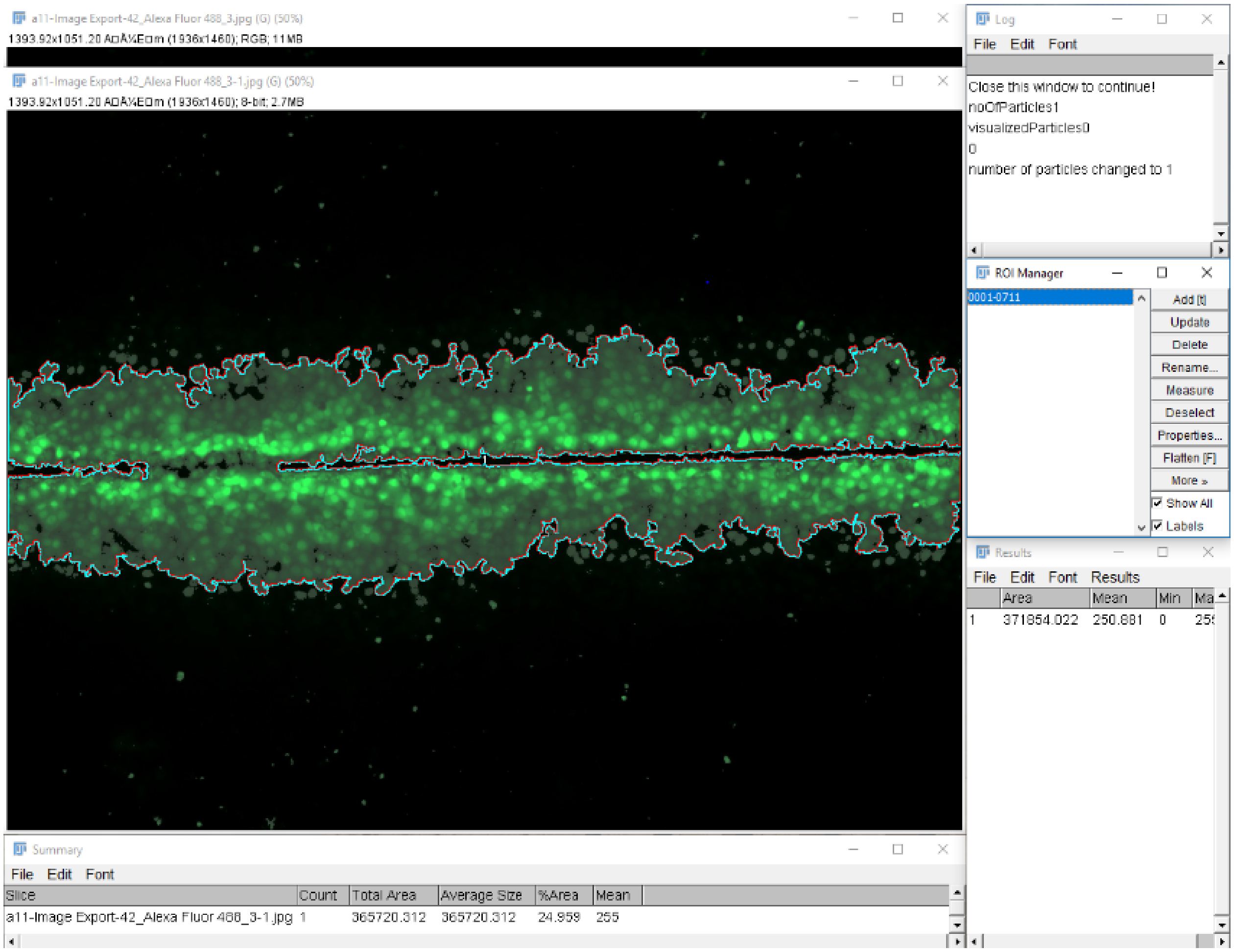

4.A Macro detected desired areas or particles -> close Log window and next image will be opened

4.B You are not satisfied with detected areas or number of particles and want to delete some -> in ROI press number belongs to the areas or particles -> DELETE-> close Log window

4.C You are not satisfied with detected areas or number of particles and want to add some -> choose shape of "heart" in FIJI toolbar and outline the desired new area or particle(s) -> ROI-> press ADD-> close Log window

Caution: there has to be at least one detected particle on the image, or analysis will stop. So first add new one and then delete another one.

5. End of analysis

Log window inform you that analysis is completed-> Close this window.

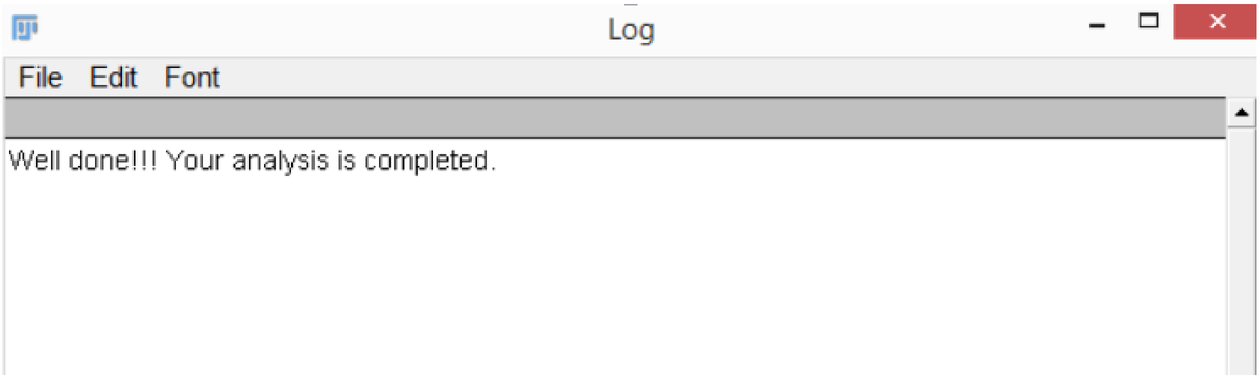

6. Outcome

The Excel file with the results is automatically saved in the folder with input images. Each excel file contains name of image, the number of particles and the sum of particles area. The “Control” file with original images interspersed with analysed images is created in the input folder. Images in the “Control” file are saved with the same name as original image.

Supplementary Text S2. Manual Analysis of Cell Count

First, open an image in FIJI. In toolbar select *Analyse* -> *Tools* -> *Grid*. Specify the grid type (lines, crosses or points), the area per point (in pixels or physical units; for the image of 1936×1460 pix: 58,500), and the colour. Tick *Bold* and *Center grid on image*. For counting, use *Multiple-point* tool (label with red on the image below) from the toolbar. Count at least 3 randomly selected squares. When counting is finished, press key *M* and multiple result by total number of squares.

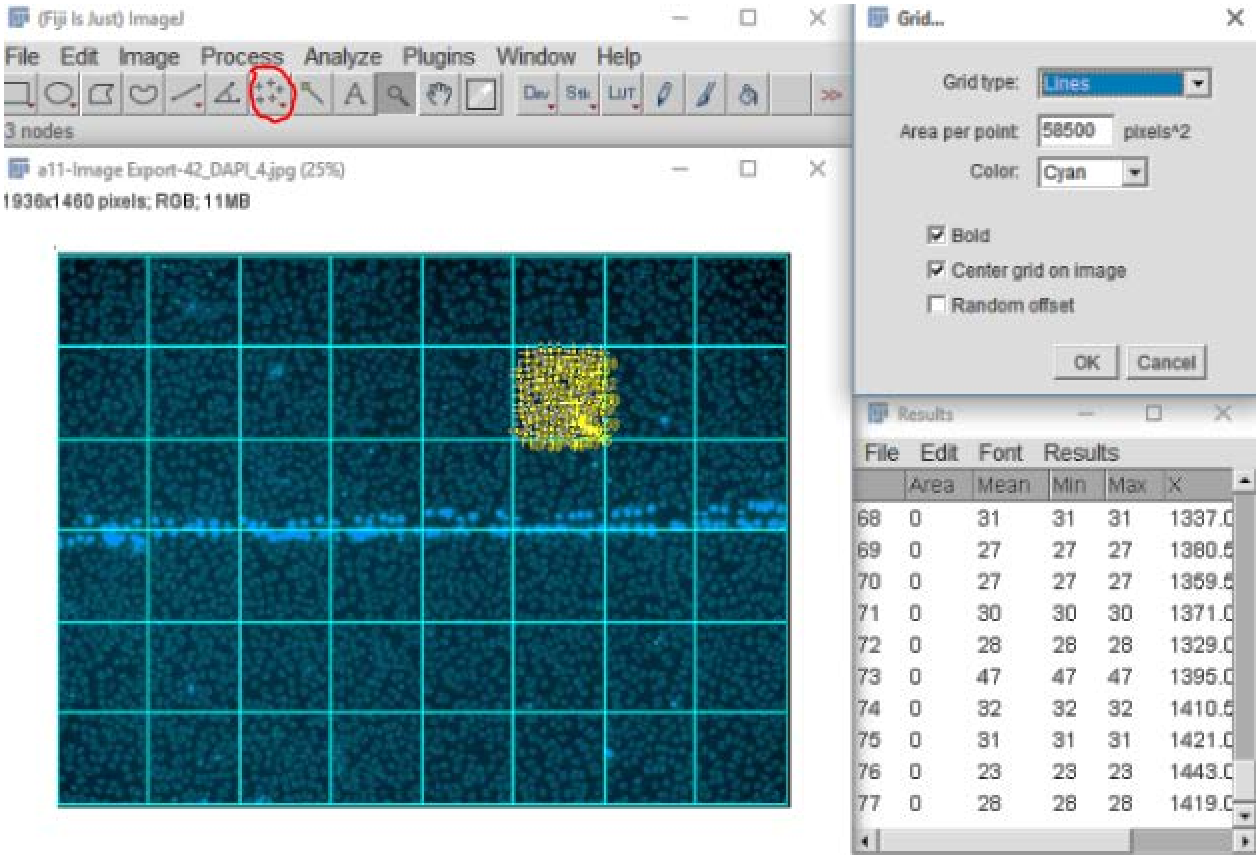

(Fiji Is Just) ImageJ
File Edit Image Process Analyze Plugins Window Help
3 nodes
a11-Image Export-42_DAPI_4.jpg (25%)
1936x1460 pixels; RGB; 11MB
Grid...
Grid type: Lines
Area per point: 58500 pixels^2
Color: Cyan
Bold
Center grid on image
Random offset
OK Cancel
Results
File Edit Font Results
Area Mean Min Max X
68 0 31 31 31 1337.0
69 0 27 27 27 1380.5
70 0 27 27 27 1359.5
71 0 30 30 30 1371.0
72 0 28 28 28 1329.0
73 0 47 47 47 1395.0
74 0 32 32 32 1410.5
75 0 31 31 31 1421.0
76 0 23 23 23 1443.0
77 0 28 28 28 1419.0

SUPPLEMENTARY TABLES

Supplementary Table S1 The comparison of the cost and usage of propidium iodide (PI) and Texas-red dextran (TRD)

**Dye**	**PI**	**TRD**
**Company**	Sigma Aldrich	Thermo Fisher
**Cat. No**	P4170	D1863
**Price per 1 mg (€)**	5.4	15.5
**Final con. (mg/mL)**	0.01	5
**Reusing**[a]	Yes	No

[a]Propidium iodide, Lucifer Yellow and Hoechst 33342 solution can be re-used at least 10times without any effect on the chemical response, while rapid fading of the signal of TRD was observed when recycled. Note: We observed that Lucifer Yellow and Hoechst 33342 are much less prone to fading than propidium iodide. Thus, when propidium signal weakens, the life-time of the dye solution can be prolonged by re-adding propidium iodide (up to 5-times, i.e. to total nominal concentration 50 µg/mL), without any significant effects on the assay performance.

Supplementary Table S2 The comparison of the speed of manual analysis and (semi)-automatic analysis in sec per image

**Endpoint**	**Manual (s)**	**Macro (s)**
GJIC/loaded cells	3-5	0.5-1
Viability	150-300	3-6
Density	150-300	3-6

Supplementary Table S3 HTS and/or HCA/HCS compatible gap junctional intercellular communication (GJIC) assays

Method	Tracer	Cell line	Miniaturization	HTS	HCA/HCS	Image/Data Analysis	Compounds	Advantages	Limitation
Cx43 GJ aequorin assay [1]	Calcium	HeLa<br>CHO<br>HEK293<br>U2OS (transduced with Cx43 and cytoAeq/α1-ARs or TRPV1)	384-well plate	*Platform ready for automated cell seeding and compound adding	*Luminescence plate reader - Lumilux Cellular Screening Platform (Perkin Elmer)	*Luminiscence-based evaluation	*<u>GJ blockers</u>: AGA	*HTS screening assay<br>*non-invasive<br>*does not require complex equipment and analysis<br>*miniaturization	*transduced donor cells co-expressing Cx43 and α1-ARs (or TRPV1) and transduced recipient cells co-expressing Cx43 and cytoAeq needed<br>* involves trypsinisation and formation of GJ channels between donor and recipient cells, loading with cytoAeq- cofactor and use of α1-ARs/TRPV1 agonists<br>*high probability of false-positive hits<br>*optimized only for Cx43
Electroporation technique [2]	Lucifer yellow	Cx43-C6	Electroporation slides	*Low through put	*Manual live cell imaging - light and fluorescence microscopy	*Semiautomatic - custom written scripts in Matlab program (not on-line available anymore)	*<u>GJ blockers</u>: CBX, Gap27, octanol, AGA	*a rapid and objective quantification with a high degree of reproducibility<br>*applicable to a large variety of adherent cell types	*a specialized electroporation equipment needed<br>*unsuitable for poorly adherent cells<br>*cell health concerns<br>*not useful for Cx channels that exclude Lucifer Yellow
Gold nanoparticle-mediated laser perforation/dye transfer (GNOME-LP/DT) [3]	Lucifer Yellow	GM-7373<br>RBE4	24-well plate	*Low through put	*Automatic fixed cell imaging - Eclipse Ti microscope (Nikon)	*Semiautomatic - a java-based ImageJ-plugin for dye diffusion plot profiles<br>*A home-made MATLAB-based software for calculation of the dye diffusion distance	*<u>GJ inhibitors:</u> CBX<br>*<u>GJ stimulators:</u> forskoline	*a high-throughput automated analysis<br>*non-invasive<br>*applicable for 3D cultivated cells or tissue	*a specialized microscopic equipment needed<br>*not useful for Cx channels that exclude Lucifer Yellow<br>*inappropriate to investigate cell assemblies or low-density cultures
Innovative scrape loading/dye transfer assay [4]	Lucifer Yellow	WB-F344<br>WB-F344-ras<br>HBE1<br>BEAS-2B<br>TM3<br>TM4	24-96-well plate	*Platform ready for automated cell seeding or compound adding	*Automatic fixed cell imaging - a fully motorized Axio Observer Z1 fluorescent microscope	*Semiautomatic - in-house made Multiassay macros created in Fiji	*<u>GJ inhibitors:</u> TPA, BGA, fluoranthene, methanol<br>*<u>GJ stimulators</u>: CAPE, sodium butyrate	* fast and simple simultaneous assessment of GJIC, cell density and viability in a large number of cells<br>*automated image acquisition and analysis<br>*no specialized equipment or technical skills needed	*invasive<br>*only for cells growing in high densities and forming nearly confluent monolayers<br>*not useful for Cx channels that exclude Lucifer Yellow
$I^{-}YFP^{QL}$ assay [5]	Iodide	LN215<br>HOS (transduced with iodide transporter/ iodide	96-well plate	*Platform ready for automated cell seeding or compound adding	*Fluorescent plate reader - POLARstar microplate reader (BMG Labtech)	*Fluori-metric evaluation	*<u>GJ blockers</u>: CBX, 2-APB<br>*40 compounds screened	*a high-throughput screening assay<br>*short assay time (10 s)<br>*non-invasive<br>*does not require complex	*specific models of donor cells (transduced with iodide transporter SLC26A4) and acceptor cells (transduced with iodide sensor protein

		sensor protein)						equipment and analysis<br>*miniaturization	H148Q/I152L) needed<br>*high probability of false-positive hits
Microfluidic GJ assay	CFDA [6]	NRK-49F	Chip	*Microfluidic platform	*Automatic live cell imaging - Axiovert 200M Zeiss	*Manual	*GJ blockers: 1-heptanol, 2-APB	*non-invasive<br>*in situ monitoring of GJIC<br>*rapid screening<br>*applicable for studying kinetics of GJCs diffusion	*a specialized microfluidic equipment needed<br>*nonspecific dye transfer of CFDA [8]<br>*a limited number of studied cells
	Calcein [7]	C6<br>Cx43-C6<br>HeLa			*Automatic live cell imaging - AxioObserver Z1 microscope Zeiss		*GJ blockers: AGA	*non-invasive<br>*ease-of-use<br>*in situ monitoring of GJIC<br>*rapid screening<br>*applicable for studying kinetics of GJCs diffusion<br>*can be scaled up for high-throughput applications<br>*low reagent consumption	*a specialized microfluidic equipment needed<br>*only suitable for monolayer forming cells<br>*nonspecific dye transfer of calcein has been observed [8]<br>*Calcein can be actively pumped out by MRPs [9]<br>*a limited number of studied cells
Microinjection [10]	HPTS	HeLa<br>HEK293<br>HL-1	35-mm Petri dish	*Robotic microinjection system	*Automatic live cell imaging - fully motorized fluorescent microscope Nikon Ti	*Manual		*precise and quantitative<br>*instantaneous delivery<br>*automated microinjection of a large of cells<br>*permits the correlation of morphological<br>*enables kinetic studies evaluating the transfer rate from one cell to another and functional data from individual cells	*low through-put/time consuming<br>*a specialized microinjection equipment needed<br>*invasive<br>*unsuitable for detecting effects that are either very rapid or require a continuous application of the stimulus
Parachute assay [11]	Calcein	WB-F344	96-well plate	*Platform ready for automated cell seeding or compound adding	*Automatic live cell imaging - ArrayScan V	*Commercially available software "Target Activation"	*GJ blockers: TPA ($_{3h}EC_{50}$ = 0.5 nM)<br>*cigarette smoke screening	*automated image acquisition and analysis	* relatively long (2-4 h) time-lapse image acquisition<br>*nonspecific dye transfer of calcein has been observed [8]<br>*calcein can be actively pumped out by MRPs [9]<br>* involves trypsinisation of the donor cells<br>*formation of GJ channels between donor and recipient cells during exposure
Preloading assay	Calcein	IAR20 [12]	384-well plate	*Automated cell seeding - multidrop 384 dispenser<br>*Automated compound adding - automated	*Automatic live cell imaging - IncuteCyte ZOOM	*Automatic - an ImageJ macro and Python script	*GJ blockers: α-chloridine, chlordanes, BGA ($_{4h}EC_{50}$ = 63 μM), Cx43 peptide disruptors<br>*GJ stimulators: cAMP	*the high level of automation with very few manual steps	* a special live microscopy equipment needed<br>* relatively long (2 h) time-lapse image acquisition<br>*nonspecific dye transfer of calcein has been observed [8]<br>*Calcein can be actively pumped out by MRPs [9]

	Cxs-C6 [13]	liquid-handling system	*Automatic live cell imaging - ArrayScan II system	*Automatic - algorithm developed on the ArraySan II	*GJ blockers: AGA, meclofenamic *486,000 small compounds screened		* involves trypsinisation of the pre-loaded cells *formation of GJ channels between donor and recipient cells during (or after) the exposure *Image analysis based on commercially available software [13]

REFERENCES: [1] Haq et al. 2013: Assay Drug Dev Technol 11, 93; **[2]** Hofgaard et al. 2009: Am J Physiol Regul Integr Comp Physiol 297, R243; **[3]** Begandt et al. 2015: J Bioenerg Biomembr 47, 441; **[4]** Dydowiczova et al. 2019: (This study); **[5]** Lee et al. 2015: BMC Biotechnology 15, 90; **[6]** Ye et al. 2011: Lab Chip 11, 1096; **[7]** Chen and Lee 2010; Integr Biol 2, 130 **[8]** Abbaci 2008: BioTechniques 45, 33; **[9]** Homolya et al. 1993: J Biol Chem 268, 21493; **[10]** Liu et al. 2014; **[11]** Roemer et al. 2013: Toxicol Lett 219, 248; **[12]** Dukic et al. 2017: SLAS Discov 22:77; **[13]** Li et al. 2003: J Biomol Screen 8, 489.

ABBREVIATION: 2-APB, aminoethoxydiphenyl borate; **α1-ARs**, α-1 adrenergic receptor; **AGA**, 18-a-glycyrrhetinic acid; **BEAS-2B**, human epithelial lung cells; **BGA**, 18-β-glycyrrhetinic acid; **C6**, rat glioma cells; **CAPE**, caffeic acid phenethyl ester; **cAMP**, cyclic adenosine monophosphate; **CBX**, carbenoxolone; **CFDA**, carboxyfluorescein diacetate; **CHO**, Chinese hamster ovary cells; **Cx**, connexin; **Cx C6**, C6 cells stably expressing channels of different connexins; **Cx43-C6**, C6 cells stably transfected with Cx43; **cytoAeq**, calcium-sensitive luminescent protein aequorin enhanced by codon optimization; **GM-7373**, bovine aortic endothelial cells; **HBE1**, human epithelial lung cells; **HCA**, high-content analysis; **HCS**, high-content screening; **HEK293**, human embryonic kidney 293 cells; **HeLa**, human cervical cancer cells; **HL-1**, mouse cardiac muscle cells; **HOS**, human bone osteosarcoma cells; **HPTS**, 8-hydroxypyrene-1,3,6-trisulfonic acid; **HTS**, high-throughput screening; **IAR20**, rat liver epithelial cells; **LN215**, human astroglioma cells; **MRPs**, multidrug resistance proteins; **NRK-49F**, normal rat kidney fibroblasts; **RBE4**, rat brain endothelial cells; **TM3**, mouse Leydig cells; **TM4**, mouse Sertoli cells; **TPA**, 12- O -tetradecanoylphorbol-13-acetate; **U2OS**, human bone osteosarcoma epithelial cells; **WB-F344**, rat liver epithelial cells.

SUPPLEMENTARY FIGURES

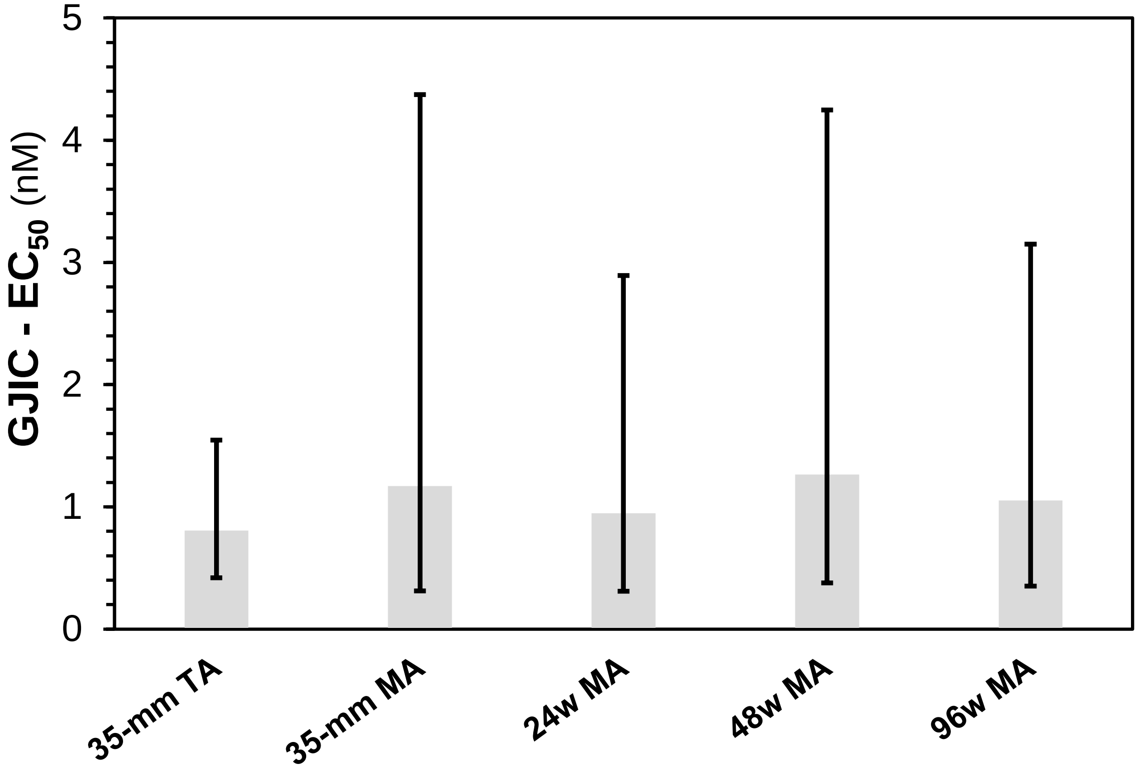

Supplementary Figure S1. The comparison of a GJIC-inhibitory activity of TPA after 1-h exposure to liver progenitor cells WB-F344 assessed in different formats of cell culture plastics (35-mm: 35-mm Petri dish; 24w: 24-well plate; 48w: 48-well plate; 96w: 96-well plate) using the traditional (TA) or innovative (MA) scrape loading-dye transfer (SL-DT) assay. The geometric mean of EC_{50} values of independent experiments (n = 3) is presented with 95% confidence interval. No significant differences among different formats (one-way ANOVA, P = 0.750).

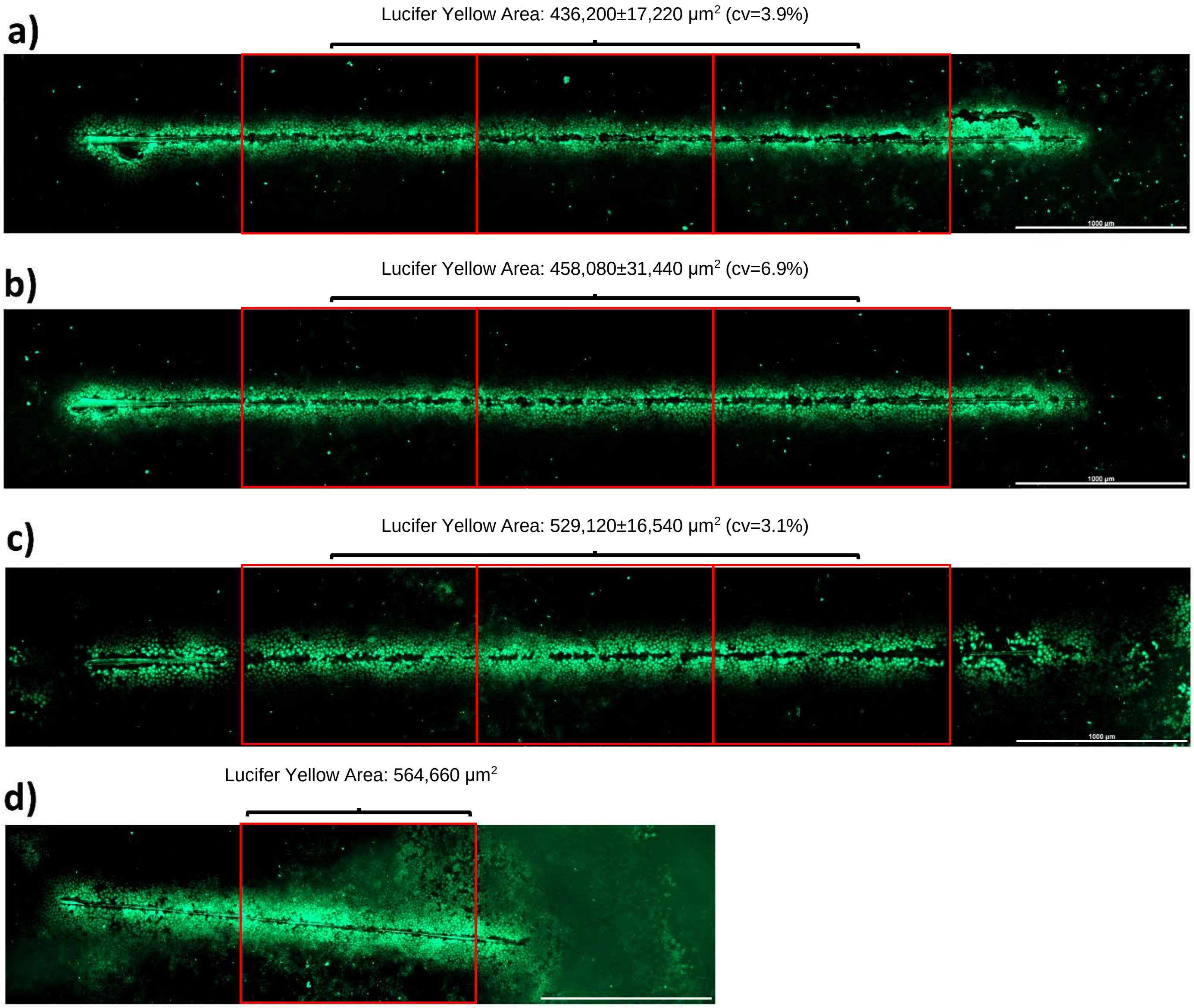

Supplementary Figure S2. Representative images obtained in the multiparametric scrape loading-dye transfer (SL-DT) assay showing the entire length of the cuts through a monolayer of non-treated liver progenitor cells WB-F344 grown in a 35-mm dish (**a**), 24-well (**b**), 48-well (**c**), and a 96-well plate (**d**). The total length of cut was app. 5,760±170 µm for steel blade #17 (**a-c**), and app. 2,770±40 µm for steel blade #66 (**d**), respectively. Composite images represent tiles of individual Field of Views (FOVs) capturing area of 1396 × 1053 µm. The average±S.D. area of cells labelled with Lucifer Yellow calculated from three individual FOVs acquired from the cut made by larger #17 blade (**a-c**) confirmed the uniformity of dye transfer along the middle part of the cut (cv<10%). Shorter length of the smaller #66 blade (**d**) allowed to capture and evaluate only a single 1396 × 1053 µm FOV from the uninterrupted middle part of the cut without including the ends of the cut or FOV overlap. Bar = 1,000 µm.

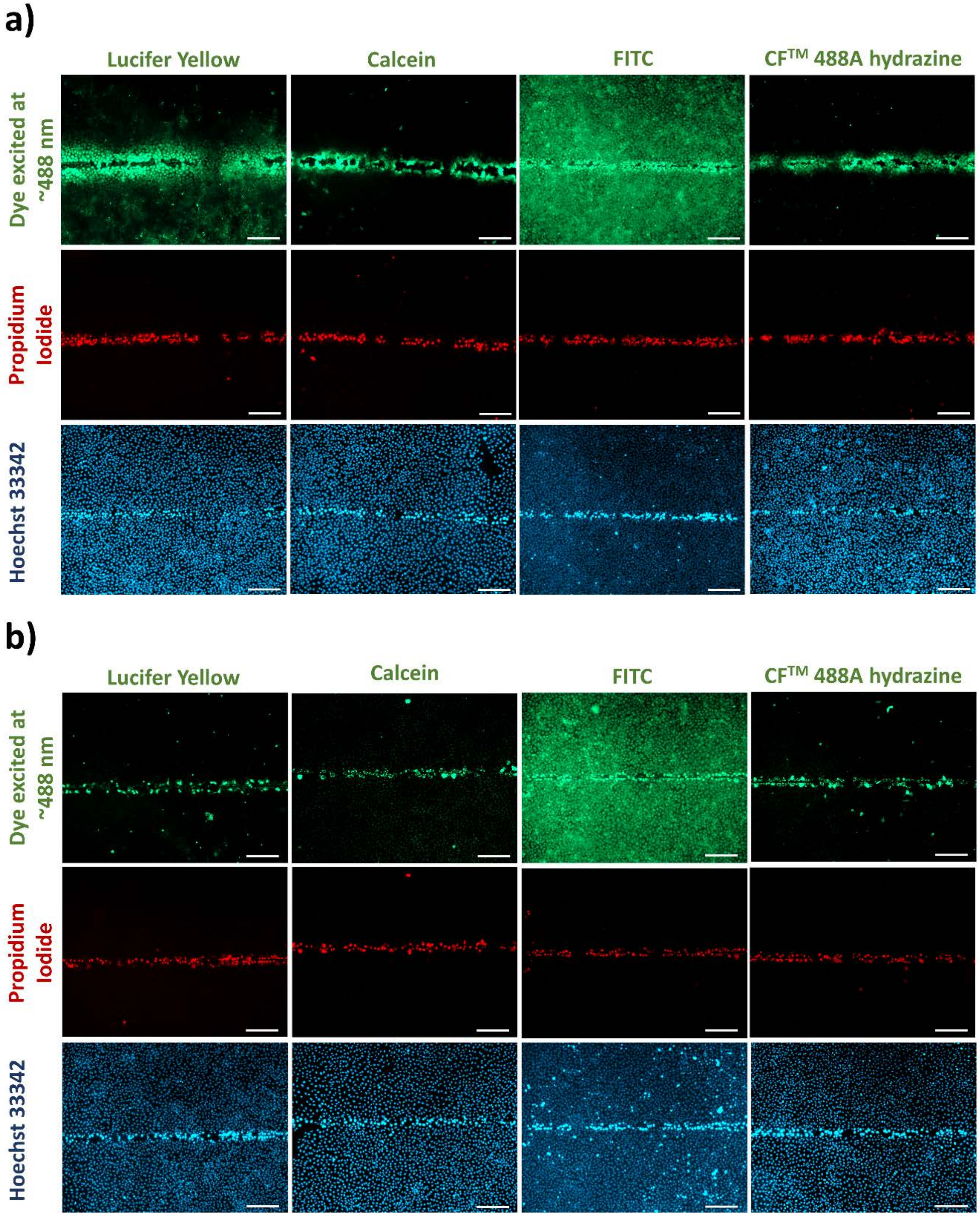

Supplementary Figure S3. Dye-loading of different types of dyes excited at ~488 nm in **(a)** not-treated and (**b**) TPA-treated (100 nM, 1 h) liver progenitor cells WB-F344 demonstrating the versatility of the multiparametric assay. FITC, fluorescein 5(6)-isothiocyanate; TPA, 12- O -tetradecanoylphorbol-13-acetate. Bar = 200 μm.

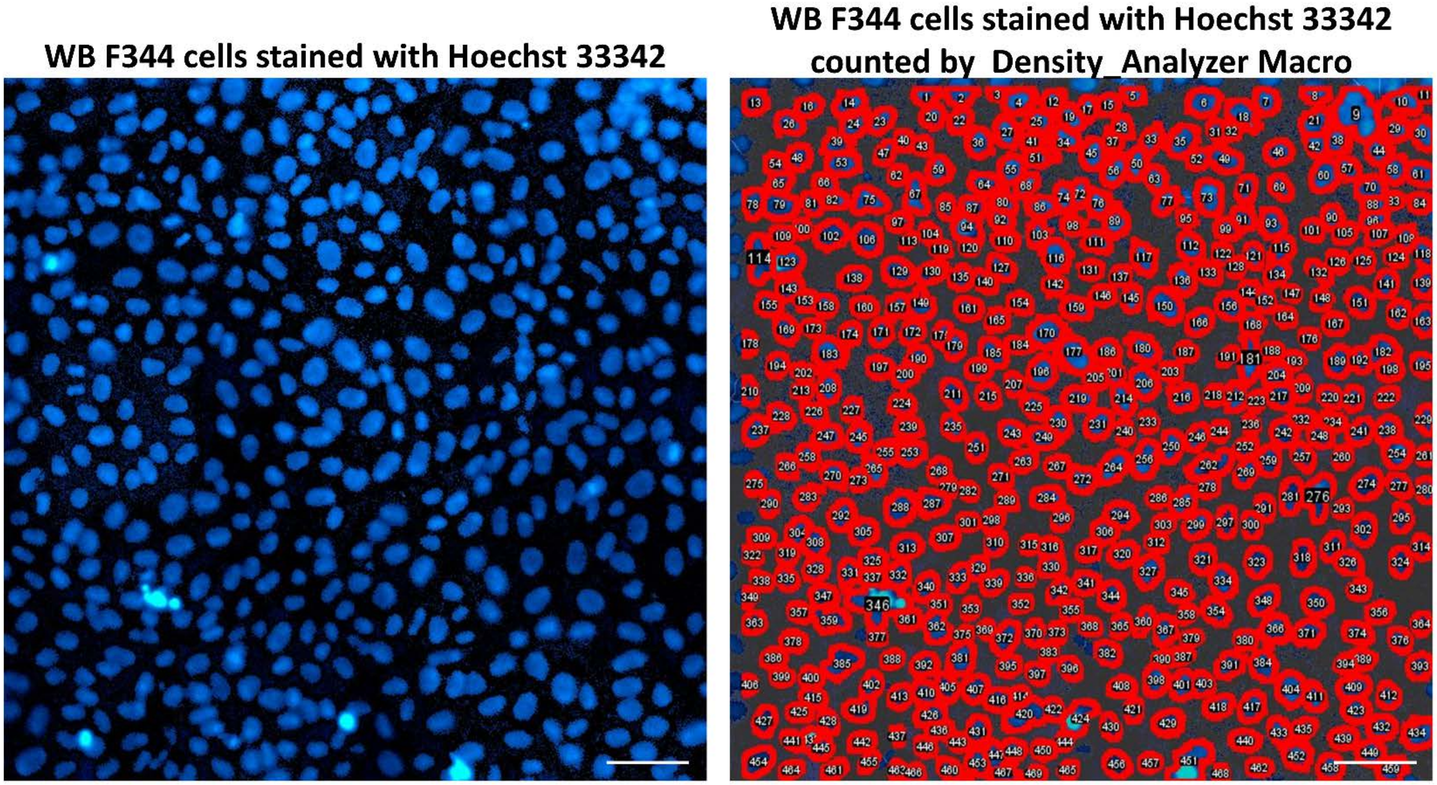

Supplementary Figure S4. Enlarged section of a representative image showing Hoechst nuclei staining in WB F344 cells (left), image segmentation and nuclei counting by the Density_Analyzer Macro (right). Bar = 50 µm.

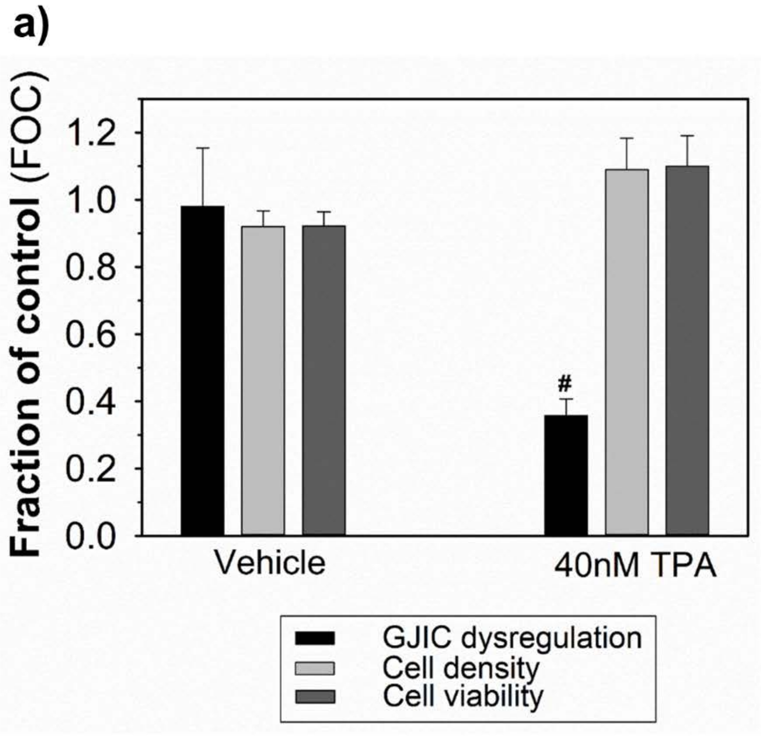

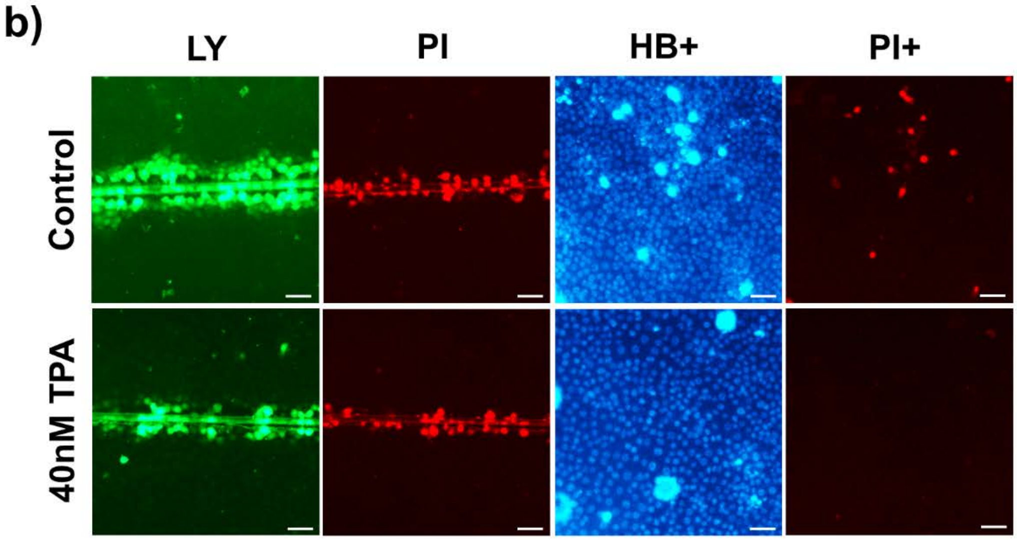

Supplementary Figure S5. The GJIC dysregulation in mouse Leydig TM3 cells induced by TPA assessed using the innovative multiparametric assay. **a)** The effects of TPA (40nM, 1-h exposure) were compared with the non-treated control and expressed as the fraction of control (FOC). Means (SD) are presented (n=3). The “#” shows significant differences (Mann–Whitney U test, the exact P = 0.002) compared to the vehicle control. **b)** The representative images of communicating cells stained with Lucifer Yellow (LY), the initial loaded cells along the cut stained with propidium iodide (PI), the total cells stained with Hoechst 33342 (HB+) and dead cells stained with PI (PI+). TPA, 12- O -tetradecanoylphorbol-13-acetate. Bar = 50 μm.

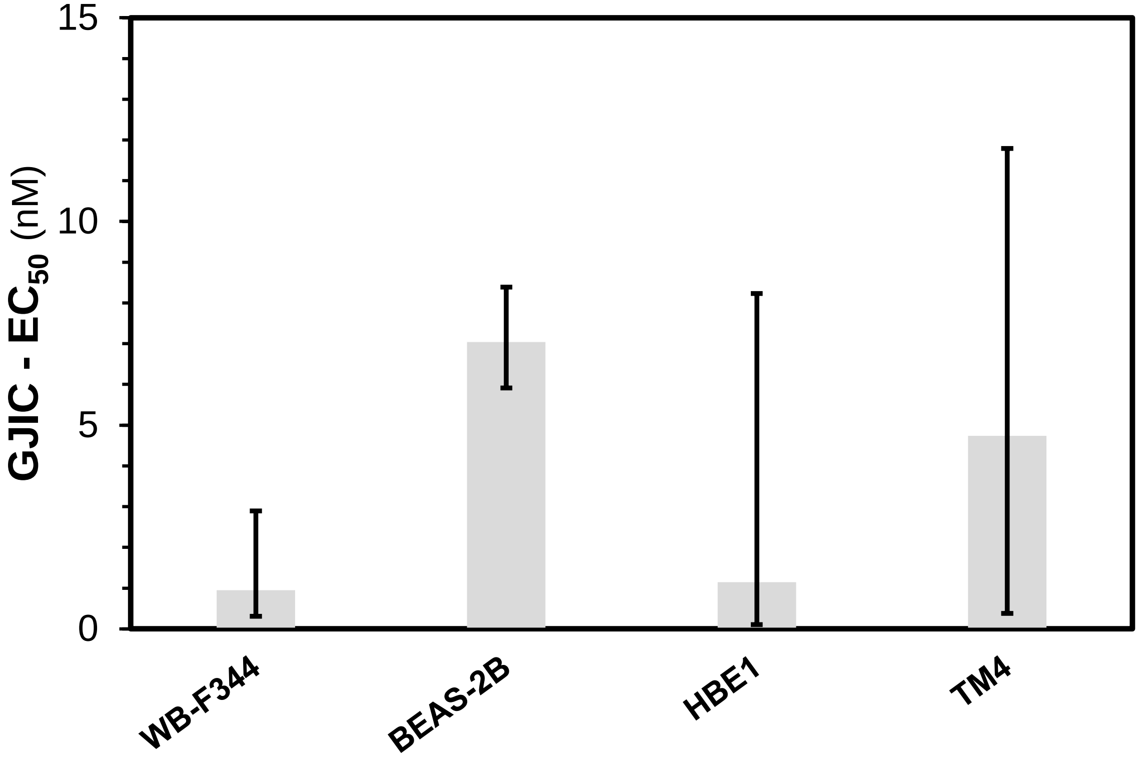

Supplementary Figure S6. The GJIC-inhibitory activity of TPA assessed using the innovative multiparametric assay (a 24-well plate format) in a variety of cell types in different cell types - WB-F344 (rat liver progenitor cells), BEAS-2B and HBE1 (human bronchial epithelial cells) and TM4 (mouse Sertoli cells). Exposure times: 1 h (WB-F344 and TM4 cells), 3 h (BEAS-2B) or 24 h (HBE1). The geometric mean of EC_{50} values of independent experiments (n = 3) is presented with 95% confidence interval.

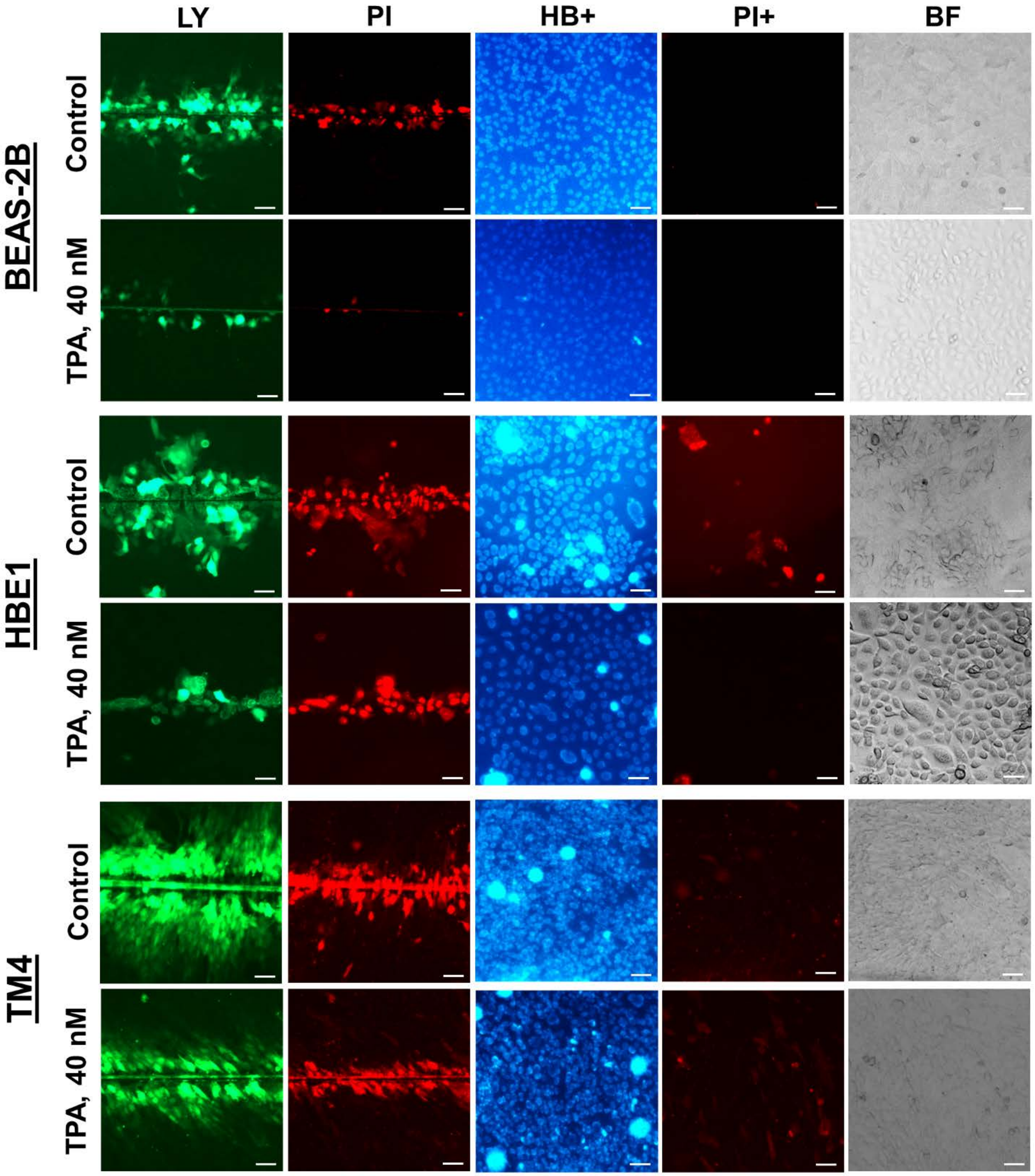

Supplementary Figure S7. The GJIC dysregulation and the effect on cell density and viability caused by TPA assessed using the innovative multiparametric assay in a variety of cell types –human lung epithelial BEAS-2B cells**,** human lung epithelial 16-HBE cells, and murine Sertoli TM4 cells. Exposure times: 1 h (WB-F344 and TM4 cells), 3 h (BEAS-2B) or 24 h (HBE1). The representative images of communicating cells stained with Lucifer Yellow (LY), the initial loaded cells along the cut stained with propidium iodide (PI), the cells stained with Hoechst 33342 (HB+) and the dead cells stained with PI (PI+). TPA, 12- O -tetradecanoylphorbol-13-acetate. Bar = 50 μm.

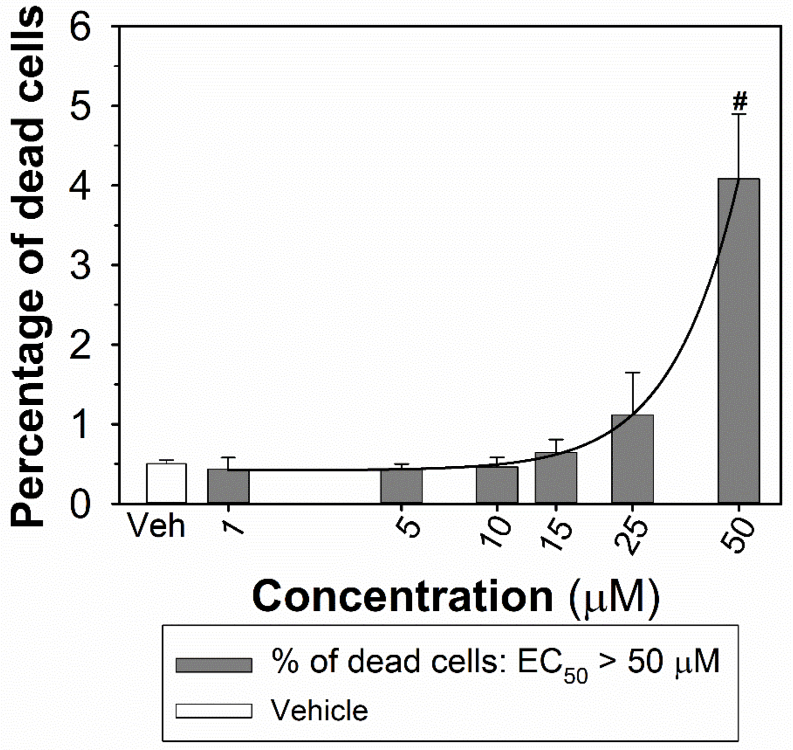

Supplementary Figure S8. The effect of caffeic acid phenethyl ester on viability of GJIC-deficient WB-F344-ras cells (ras-transformed rat liver progenitor WB-F344 cells) assessed using a multiparametric assay (a 24-well plate format, a 24-h exposure). The % of dead cells were calculated as % of propidium iodide (PI)-stained cells normalized to Hoechst 33342 (HB)-stained cells. Means (SD) of independent experiments are presented (n=3). "#" denotes significant differences at P≤0.05 compared to the vehicle control (Kruskal-Wallis ANOVA with Dunnett's test).

Appendix I

ImageJ Cut_ Analyzer Macro

```
//This macro automatically detects and measures gap junction intercellular communication or loaded cells.
//It requires user input of folder with images to be analysed and name of result table.
//Results and outcome images are automatically saved in input folder. Parameters such as maximum
//number of images, minimal size, scale are determined manually by the user into dialog menu.

// Recommended initial values
var initial_scale = 0.72;
var initial_minSize = 5000;
var initial_maxNoImgs = 500;

// Create and display dialog menu
Dialog.create("Cut_Analyzer");
Dialog.addString("Folder name", "\\", 100);
Dialog.addString("Results table name", "", 100);
Dialog.addNumber("Maximum number of images", initial_maxNoImgs);
Dialog.addNumber("Minimal size", initial_minSize);
Dialog.addNumber("Scale (um/px)", initial_scale);
Dialog.show();

// Assign values from dialog menu
gjicdir = Dialog.getString;
targetTable = Dialog.getString;
maxNoImgs = Dialog.getNumber;
minSize = Dialog.getNumber;
scale = Dialog.getNumber;

// Create control folder with processed images
File.mkdir(gjicdir + "\\control");

// Hide images
setBatchMode(false);
```

```
// Search for gjic images in gjicdir folder
gjicList = getFileList(gjicdir);

// Initialize xls file and set header
outFilePath = gjicdir + "results.xls";
headerString = "name of image\t" + "no_particles\t" + "sum_area\t";
File.saveString(headerString, outFilePath);
File.append(" ", outFilePath);

// Run script
var visualizedParticles = 0;
analyseImages();
endScript();

// Open all images and analyze them one by one
function analyseImages () {
	for (i=0; i < gjicList.length; i++) {
		if (i < maxNoImgs) {
			if (indexOf(gjicList[i], '/') == -1) {
				run("Clear Results");
				showProgress(i + 1, gjicList.length);
				gjicImage = gjicdir + gjicList[i];
			open(gjicImage);
			original = getTitle();
			open(gjicImage);

			// Process image and find particles
			run("Set Scale...", "distance=1 known=scale pixel=1 unit=ďż˝m global");
				run("8-bit");
				run("Invert");
				run("Enhance Contrast...", "saturated=5");
				run("Auto Threshold", "method=Mean");
				run("ROI Manager...");
```

```
run("Analyze Particles...", "size=" + minSize + "-5000" + "show=[Overlay Outlines] display summarize add");

// Combine original and processed images
run("Add Image...","image=[" + original + "] x=0 y=0 opacity=80");
print("Close this window to continue!");

// Wait until the user accept/edit selected areas in ROI manager
checkPause();
function checkPause(){
	if (!isOpen("Log")){
		if (roiManager("count") > 0) {
			indices = newArray();
			for (j = 0; j < roiManager("count"); j++){
				indices = Array.concat(indices, j);
			};

			noResults = nResults();
			sumArea = 0;

			for (r = 0; r < noResults; r++) {
				sumArea = sumArea + getResult("Area", r);
			}

			File.append(original + "\t" + noResults + "\t" + sumArea, outFilePath);

			highlight(indices);

			// Save output to control folder
			saveAs("Jpeg", gjicdir + "control\\" + removeExtension(gjicList[i]) + "_gjic.jpg");

			truncateROIManager();

		}
```

```
                                        run("Close All");
                                        call("java.lang.System.gc");

                                }else{
                                        wait(100);
                                        visualiseParticles();
                                        checkPause();
                                }
                        }

                }
        }
    }
}

// Clear the content of ROI manager
function truncateROIManager(){
    particlesToDelete = roiManager("count");
    if (particlesToDelete > 0 ) {
        indices = newArray();
        for (i = 0; i < particlesToDelete; i++){
            indices = Array.concat(indices, i);
        };

        roiManager("Select", indices);
        roiManager("Delete");
    }
};

// Highlight detected particles in the image
function visualiseParticles () {
    particlesCount = roiManager("count");

    if (visualizedParticles != particlesCount){
```

```
		indices = newArray();
		for (i = 0; i < particlesCount; i++){
			indices = Array.concat(indices, i);
		};

		highlight(indices);
		visualizedParticles = particlesCount;

		roiManager("Update");
		print ("number of particles changed to", visualizedParticles);

		run("Clear Results");
		roiManager("Measure");
	}
}

// Highlight given indices through the roi manager
function highlight (indices) {
	roiManager("Select", indices);
	roiManager("Set Color", "red");
	roiManager("Set Line Width", 5);
}

// Get the name of file before the extension suffix
function removeExtension (nameWith) {
	parts = split(nameWith, ".");
	return parts[0]
}

// Close all windows
function endScript () {
	selectWindow("ROI Manager");
	run("Close");
```

```
        selectWindow("Results");
        run("Close");

        print("Well done!!! Your analysis is completed.")
}
```

Appendix II

ImageJ Viability_ Analyzer Macro

//This macro automatically detects and measures dead cells in the image.

//It requires user input of folder with images to be analysed and name of result table.

//Results and outcome images are automatically saved in input folder.

//Parameters such as maximum number of images, minimal size, scale are determined manually //by the user into dialog menu

```
// Recommended initial values
var scale = 0.72;
var minSize = 1;
var maxNoImgs = 500;
var visualizedParticles = 0;

// Create and display dialog menu
Dialog.create("Viability_Analyzer");
Dialog.addString("Folder name", "\\", 100);
Dialog.addString("Results table name", "", 100);
Dialog.addNumber("Maximum number of images", maxNoImgs);
Dialog.addNumber("Minimal size", minSize);
Dialog.addNumber("Scale (um/px)", scale);
Dialog.show();

// Assign values from dialog menu
viabilitydir = Dialog.getString;
targetTable = Dialog.getString;
maxNoImgs = Dialog.getNumber;
minSize = Dialog.getNumber;
scale = Dialog.getNumber;

// Create control folder with processed images
File.mkdir(viabilitydir + "\\control");

// Hide images
setBatchMode(false);
```

```
// Search for images in input folder
viabilityList = getFileList(viabilitydir);

viabilityAreas = newArray();

// Initialize xls file and set header
outFilePath = viabilitydir + "results.xls";
headerString = "name of image\t" + "no_particles\t" + "sum_area\t";
File.saveString(headerString, outFilePath);
File.append(" ", outFilePath);

// Run script
analyseImages();
saveResults();
endScript();

// Open all images and analyze them one by one
function analyseImages () {
	for (i=0; i<viabilityList.length; i++) {
		if (i < maxNoImgs) {
			if (indexOf(viabilityList[i], '/') == -1) {
				run("Clear Results");
				showProgress(i+1, viabilityList.length);
				viabilityImage = viabilitydir + viabilityList[i];
			open(viabilityImage);
			original=getTitle();
			open(viabilityImage);

 // Process image and find particles in upper part above the cut
			run("Set Scale...", "distance=1 known=scale pixel=1 unit=ďż˝m global");
				run("Enhance Contrast", "saturated=0.05");
				run("8-bit");
				run("Auto Threshold", "method=Otsu white");
				makeRectangle(4, 4, 1930, 558);
				run("ROI Manager...");
				run("Analyze Particles...", "size="+ minSize + "-5000" + "show=[Overlay Outlines]
display summarize add");
```

```
// Open image again
                                        open(viabilityImage);

// Process image and find particles in the bottom section under the cut
                                  run("Set Scale...", "distance=1 known=scale pixel=1 unit=ďż˝m global");
                                        run("Enhance Contrast", "saturated=0.05");
                                        run("8-bit");
                                        run("Auto Threshold", "method=Otsu white");
                                        makeRectangle(3, 921, 1929, 531);
                                        run("ROI Manager...");
                                        run("Analyze Particles...", "size="+ minSize + "-5000" + "show=[Overlay Outlines]
display summarize add");

// Combine original and processed images
                                        run("Add Image...","image=["+original+"] x=0 y=0 opacity=80");
                                        print("Close this window to continue!");

// Wait until the user accept/edit selected areas in ROI manager
                                        checkPause();
                                        function checkPause(){
                                                if (!isOpen("Log")){


                                                        indices = newArray();
                                                        for (j = 0; j< roiManager("count"); j++){
                                                                indices = Array.concat(indices, j);
                                                        };

                                                        noResults = nResults();
                                                        sum_area = 0;

                                                        for (r = 0; r < noResults; r++) {
                                                                sum_area = sum_area + getResult("Area",r);

                                                        }
```

```
                        File.append(original + "\t" + noResults + "\t" + sum_area,
outFilePath);

                        roiManager("Select", indices);
                        roiManager("Set Color", "red");
                        roiManager("Set Line Width", 5);

// Save output to control folder
                        saveAs("Jpeg", viabilitydir + "control\\" +
removeExtension(viabilityList[i]) + "_viability.jpg");



                        truncateROIManager();

                        run("Close All");

                        call("java.lang.System.gc");

                    }else{
                        wait(100);
                        visualiseParticles();
                        checkPause();
                    }
                }

            }
        }
    }
}

// Clear the content of ROI manager
function truncateROIManager(){
    particlesToDelete = roiManager("count");
    if (particlesToDelete > 0 ) {
        indices = newArray();
        for (i = 0; i< particlesToDelete; i++){
```

```
                indices = Array.concat(indices, i);
            };

            roiManager("Select", indices);
            roiManager("Delete");
        }

};



function saveResults () {
        selectWindow("Summary");

}

// Get the name of file before the extension suffix
function removeExtension (nameWith) {
        parts=split(nameWith, ".");
        return parts[0]
}

// Close all windows
function endScript () {
        selectWindow("ROI Manager");
        run("Close");

        selectWindow("Results");
        run("Close");

        print("Well done!!! Your analysis is completed.")
};

// Highlight detected particles in the image
function visualiseParticles () {

        particlesCount = roiManager("count");
```

```
	if (visualizedParticles != particlesCount ){
		print("noOfParticles" + particlesCount);
		print("visualizedParticles" + visualizedParticles);

		indices = newArray();
		for (i = 0; i< particlesCount; i++){
			indices = Array.concat(indices, i);
		};
		Array.print(indices);

// Highlight given indices through the roi manager
		roiManager("Select", indices);
		roiManager("Set Color", "red");
		roiManager("Set Line Width", 5);
		visualizedParticles = particlesCount;

		roiManager("Update");
		print ("number of particles changed to",  visualizedParticles);

		run("Clear Results");
		roiManager("Measure");

	}

}
```

Appendix III

ImageJ Density_ Analyzer Macro

```
//This macro automatically detects and measures cell density.
//It requires user input of folder with images to be analysed and name of result table.
//Results and outcome images are automatically saved in input folder. Parameters such as maximum
//number of images, minimal size, scale are determined manually by the user into dialog menu.

// Recommended initial values
var scale = 0.72;
var minSize = 1;
var maxNoImgs = 500;
var visualizedParticles = 0;

// Create and display dialog menu
Dialog.create("Density_Analyzer");
Dialog.addString("Folder name", "\\", 100);
Dialog.addString("Results table name", "Fun with density", 100);
Dialog.addNumber("Maximum number of images", maxNoImgs);
Dialog.addNumber("Minimal size", minSize);
Dialog.addNumber("Scale (um/px)", scale);
Dialog.show();

// Assign values from dialog menu
densitydir = Dialog.getString;
targetTable = Dialog.getString;
maxNoImgs = Dialog.getNumber;
minSize = Dialog.getNumber;
scale = Dialog.getNumber;

//Create control folder with processed images
File.mkdir(densitydir + "\\control");

//Hide images
```

```
setBatchMode(false);

//Search for images in input folder
densityList = getFileList(densitydir);

densityAreas = newArray();

//Initialize xls file and set header
outFilePath = densitydir + "results.xls";
headerString = "name of image\t" + "no_particles\t" + "sum_area\t";
File.saveString(headerString, outFilePath);
File.append(" ", outFilePath);

// Run script
analyseImages();
saveResults();
endScript();

//Open all images and analyze them one by one
function analyseImages () {
	for (i=0; i<densityList.length; i++) {
		if (i < maxNoImgs) {
			if (indexOf(densityList[i], '/') == -1) {
				run("Clear Results");
				showProgress(i+1, densityList.length);
				densityImage = densitydir + densityList[i];
			open(densityImage);
			original=getTitle();
			open(densityImage);

			// Process image and find particles in upper part above the cut
			run("Set Scale...", "distance=1 known=scale pixel=1 unit=ďż˝m global");
				run("Enhance Contrast", "saturated=0.5");
				run("16-bit");
```

```
		run("Auto Threshold", "method=Mean dark");
		run("Make Binary");
		run("Watershed");
		makeRectangle(12, 9, 1923, 681);
		run("ROI Manager...");
		run("Analyze Particles...", "size="+ minSize + "-5000" + "show=[Overlay Outlines]
display summarize add");

		//Open image again
		open(densityImage);

		// Process image and find particles in the bottom section under the cut
	run("Set Scale...", "distance=1 known=scale pixel=1 unit=ďż˝m global");
		run("Enhance Contrast", "saturated=0.5");
		run("16-bit");
		run("Auto Threshold", "method=Mean dark");
		run("Make Binary");
		run("Watershed");
		makeRectangle(3, 921, 1929, 531);
		run("ROI Manager...");
		run("Analyze Particles...", "size="+ minSize + "-5000" + "show=[Overlay Outlines]
display summarize add");

		// Combine original and processed images
		run("Add Image...","image=["+original+"] x=0 y=0 opacity=80");
		print("Close this window to continue!");

		// Wait until the user accept/edit selected areas in ROI manager
		checkPause();
		function checkPause(){
			if (!isOpen("Log")){
				indices = newArray();
				for (j = 0; j< roiManager("count"); j++){
					indices = Array.concat(indices, j);
```

```
						};

						noResults = nResults();
						sum_area = 0;
						for (r = 0; r < noResults; r++) {
							sum_area = sum_area + getResult("Area",r);
						}
						File.append(original + "\t" + noResults + "\t" + sum_area, 
outFilePath);

						roiManager("Select", indices);
						roiManager("Set Color", "red");
						roiManager("Set Line Width", 5);

						//Save output to control folder
						saveAs("Jpeg", densitydir + "control\\" + 
removeExtension(densityList[i]) + "_density.jpg");
						truncateROIManager();

						run("Close All");
						call("java.lang.System.gc");

					}else{
						wait(100);
						visualiseParticles();
						checkPause();
					}
				}

			}
		}
	}
}
```

```
// Clear the content of ROI manager
function truncateROIManager(){
	particlesToDelete = roiManager("count");
	if (particlesToDelete > 0 ) {
		indices = newArray();
		for (i = 0; i< particlesToDelete; i++){
			indices = Array.concat(indices, i);
		};

		roiManager("Select", indices);
		roiManager("Delete");
	}

};



function saveResults () {
	selectWindow("Summary");
}

// Get the name of file before the extension suffix
function removeExtension (nameWith) {
	parts=split(nameWith, ".");
	return parts[0]
}

// Close all windows
function endScript () {
	selectWindow("ROI Manager");
	run("Close");

	selectWindow("Results");
	run("Close");
```

```
	print("Well done!!! Your analysis is completed.")
};

// Highlight detected particles in the image
function visualiseParticles () {
	particlesCount = roiManager("count");

	if (visualizedParticles != particlesCount ){
		print("noOfParticles" + particlesCount);
		print("visualizedParticles" + visualizedParticles);

		indices = newArray();
		for (i = 0; i< particlesCount; i++){
			indices = Array.concat(indices, i);
		};

	// Highlight given indices through the roi manager
		Array.print(indices);
		roiManager("Select", indices);
		roiManager("Set Color", "red");
		roiManager("Set Line Width", 5);
		visualizedParticles = particlesCount;
		roiManager("Update");
		print ("number of particles changed to",  visualizedParticles);
		run("Clear Results");
		roiManager("Measure");
	}

}
```

Appendix IV

https://1drv.ms/f/s!Aj3F6lRs5K_mn10TfvMs1crQBT6V
